# Particle Morphology Controls the Bulk Mechanical Behavior of Far-Side Lunar Regolith from Chang’e-6 Samples and Deep Learning

**DOI:** 10.34133/research.1064

**Published:** 2026-01-08

**Authors:** Hao Wang, Siqi Zhou, Xue Zhang, Qi Zhou, Yu Jiang, Yutong Deng, Jiayuan Liu, Zhiqian Lin, Feng Li, Chi Zhang, Wei Yang, Chao Wang, Xiaohua Tong

**Affiliations:** ^1^ School of Transportation Science and Engineering, Beihang University, Beijing 100191, China.; ^2^ Department of Civil and Environmental Engineering, University of Liverpool, Liverpool L69 3BX, UK.; ^3^ School of Future Aerospace Technology, Beihang University, Beijing 100191, China.; ^4^ Key Laboratory of Earth and Planetary Physics, Institute of Geology and Geophysics, Chinese Academy of Sciences, Beijing 100029, China.; ^5^ College of Surveying and Geo-informatics, Tongji University, Shanghai 200092, China.

## Abstract

Lunar far-side samples returned by the Chang’e-6 mission offer unprecedented insights into regolith properties within the South Pole–Aitken basin, essential for advancing lunar exploration and in situ resource utilization. This paper presents an integrated characterization framework combining high-resolution x-ray micro-computed tomography with semisupervised machine learning to reconstruct and analyze 349,740 individual particles at high throughput. Morphological analysis demonstrates that far-side regolith exhibits greater irregularity than previously characterized near-side samples, with a median particle diameter of 60.51 μm and a mean 3-dimensional sphericity of 0.74—values distinct from those reported for Apollo and Chang’e-5 materials. Discrete element method simulations incorporating these high-fidelity morphologies under representative lunar surface confining pressures (5 to 15 kPa) reveal a high internal friction angle of 47.96° and a cohesion of 1.08 kPa. These parameters exceed Surveyor mission estimates and align with the upper range of Apollo program values, indicating enhanced mechanical strength and cohesion in far-side regolith. The superior mechanical properties arise primarily from pronounced particle irregularity promoting strong mechanical interlocking, potentially augmented by cementation from abundant glassy agglutinate phases. These findings establish critical geotechnical benchmarks for lunar far-side materials, providing essential design parameters for future robotic and crewed missions, landing site selection, and infrastructure development.

## Introduction

The Chang’e-6 (CE-6) mission achieved a historic milestone by successfully returning the first lunar far-side samples to Earth [1]. The mission targeted the South Pole–Aitken (SPA) basin, the Moon’s largest, oldest, and deepest confirmed impact structure [2]. This unique geological setting provides an unprecedented opportunity to investigate the Moon’s early evolution, as it potentially exposes deep crustal materials or even upper mantle components that remain inaccessible on the near side [3]. Beyond its scientific significance, the SPA basin’s strategic location—near potential water-ice deposits and regions of extended sunlight—also makes it a prime candidate for future long-term exploration missions, including the proposed International Lunar Research Station [4,5].

The success of these future missions critically depends on understanding the engineering properties of the local regolith. Geotechnical characteristics, particularly bearing capacity and shear strength, fundamentally influence surface operations including landing system design [6], rover mobility [7], habitat construction [8], and in situ resource utilization strategies [9–11]. Comprehensive mechanical characterization of far-side regolith is therefore essential for safe and efficient future exploration in this region.

Direct mechanical testing of returned lunar samples faces substantial constraints. The material’s exceptional scarcity precludes the large-scale destructive tests required by conventional geotechnical engineering [1,12]. Additionally, replicating extreme lunar surface conditions (i.e., high vacuum, large thermal gradients, and reduced gravity) in terrestrial laboratories presents substantial challenges [13]. Given these limitations, the discrete element method (DEM) has emerged as an indispensable computational tool. By simulating the interactions of individual particles based on microscale physical laws, DEM enables virtual experiments that predict bulk mechanical behavior, thus circumventing the constraints of physical testing [14–16].

The accuracy of DEM simulations critically depends on the fidelity of microscale input parameters [17,18]. Three-dimensional (3D) particle morphology is particularly crucial, as size, shape, and roughness govern interparticle contact mechanisms, packing density, and frictional properties that collectively determine bulk shear strength. Beyond engineering applications, particle morphology serves as a physical archive of regolith history [19,20]. Grain shapes and textures encode information about their origin and exposure to space weathering—the suite of processes affecting the lunar surface, including micrometeorite impacts that cause particle size reduction and the formation of glassy agglutinates (microscopic particles bonded together by impact-melted glass) [21]. The cumulative effect of these processes determines the regolith’s maturity, where higher maturity corresponds to finer grain sizes and greater abundance of agglutinates [5]. High-fidelity 3D morphological characterization thus serves dual purposes: providing essential data for accurate mechanical modeling while offering insights into geological processes that shaped the lunar far side.

To contextualize far-side material properties, it is instructive to consider previously studied near-side samples. Comprehensive analysis of 143 Apollo and Luna samples by NASA [22] revealed that near-side regolith is a well-graded silty sand with a median particle diameter (*d*_50_) typically ranging from 46 to 110 μm, averaging approximately 72 μm [23]. The mechanical properties showed pronounced variability depending on measurement methods and conditions. In situ estimates from Surveyor 3 and 7 lander data suggested cohesion (*c*) values of 0.35 to 0.7 kPa and internal friction angles (*φ*) of 35° to 37° [24]. Apollo mission analyses generally placed *c* between 0.1 and 1.0 kPa and *φ* between 30° and 50° for near-surface conditions [25], with both properties known to increase markedly with depth due to rising density. Laboratory tests on Soviet Luna 16 and 20 samples yielded considerably lower *φ* (20° to 25°) [5], although these were measured under high confining pressures not representative of lunar surface conditions. Recent Chang’e-5 (CE-5) studies have highlighted the influence of measurement techniques on morphological parameters. Two-dimensional (2D) optical microscopy reported a *d*_50_ of approximately 53 μm [12], while laser diffraction analysis yielded a *d*_50_ of about 55 μm [26]. These values are notably finer than the 58- to 64-μm range obtained from 3D x-ray micro-computed tomography (micro-CT) analyses [27,28], demonstrating systematic variations between methodologies. Three-dimensional analyses of CE-5 samples yielded a mean sphericity (*SPH*) of ~0.87 and an aspect ratio (*AR*) of ~0.55, indicating greater irregularity compared to that of Apollo and Luna samples (*AR* ~ 0.58) [27–29]. Given the unique geological context of the CE-6 landing site, a critical question emerges: does far-side regolith share these near-side characteristics or possess distinct properties requiring special consideration in future mission planning?

Acquiring a comprehensive 3D morphological dataset for precious extraterrestrial materials requires advanced, nondestructive techniques. Traditional bulk methods face critical limitations: sieve analysis is restricted to particles larger than 75 μm and is biased by shape [30], while sedimentation analysis risks irreversible sample loss, making it unsuitable for precious specimens [31]. Two-dimensional imaging techniques, although widely used, fail to capture true 3D grain geometry, as sectional profiles can yield substantially different morphological attributes depending on the viewing angle [1,12,26]. Micro-CT technology has therefore emerged as the optimal analytical approach, offering high-resolution 3D imaging without sample damage [32]. However, extracting accurate information from lunar regolith CT images presents substantial challenges, as low overall contrast, wide particle size ranges, and complex grain structures render traditional segmentation methods ineffective. Advanced computational pipelines are therefore necessary. While CE-5 sample analysis successfully employed machine learning classifiers such as random forest (RF) [33], deep learning methods, particularly U-Net architectures that excel at learning intricate spatial features [34,35], have proven well suited for this task, enabling the high-fidelity segmentation for robust morphological analysis.

To address these challenges and investigate the properties of far-side regolith, this study presents an integrated characterization framework that systematically links microscale morphology with macroscale mechanics. The workflow begins with high-resolution micro-CT imaging and a semisupervised deep learning pipeline for the high-throughput 3D reconstruction of 349,740 individual particles, followed by morphological quantification. These morphological data then inform DEM simulations to predict the bulk shear strength parameters (i.e., *c* and *φ*) of the CE-6 regolith. This integrated approach provides benchmark geotechnical properties for this lunar environment, offering essential design parameters for future robotic and crewed missions, including the International Lunar Research Station, subsequent Chang’e missions, and the NASA Artemis program. The methodology presented here also provides a nondestructive approach for analyzing precious extraterrestrial materials, contributing to comparative planetology studies.

## Results and Discussion

The analysis connects microscale particle morphology to macroscale bulk mechanics through a systematic, multistage workflow. Results demonstrate that the highly irregular morphology of CE-6 particles is a key factor controlling the regolith’s bulk mechanical response. This irregularity promotes mechanical interlocking, which yields a high predicted shear strength, characterized by an internal friction angle of 47.96° and a cohesion of 1.08 kPa. The following sections present detailed results of the integrated workflow, from image segmentation and morphological analysis to DEM simulations that support these findings.

### Micro-CT image segmentation results

**Fig. 1.**
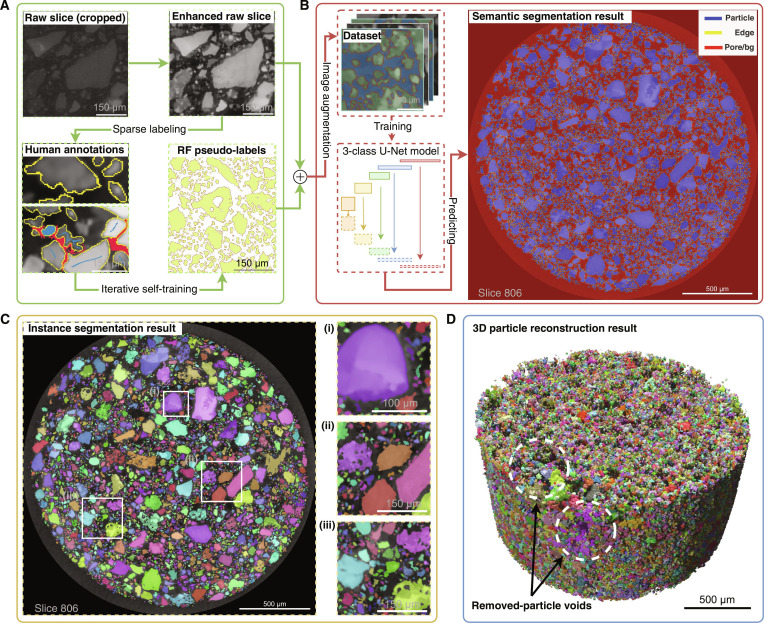
Integrated pipeline for 3-dimensional (3D) segmentation and reconstruction of Chang’e-6 (CE-6) lunar regolith particles from micro-computed tomography (micro-CT) images. (A) Pseudo-label generation workflow, starting from preprocessed slices and sparse annotations to produce random forest (RF) pseudo-labels through iterative self-training. (B) U-Net semantic segmentation, where augmented images and pseudo-labels train the model to yield 3-class predictions (particle, edge, and pore; slice 806 shown). (C) Instance segmentation results using the watershed algorithm (slice 806 shown); the zoomed-in regions demonstrate the algorithm’s robustness in various scenarios: (i) separating fine particles adhered to large grains; (ii) delineating precise boundaries between densely packed angular particles; and (iii) preserving the integrity of particles with complex internal porosity. (D) Final 3D reconstruction of the filtered particle dataset after removing incomplete and subthreshold instances [71].

To accurately characterize the complex morphology of CE-6 regolith particles, a multistage workflow was developed integrating advanced image processing, semisupervised deep learning, and instance segmentation techniques. A schematic overview of this integrated pipeline, which systematically transforms raw micro-CT slices into a final dataset of reconstructed 3D particles, is provided in Fig. 1.

The initial stage of the pipeline employed an iterative self-training framework to generate RF pseudo-labels (Fig. 1A and Fig. S1c). Subsequently, the RF-generated pseudo-labels and preprocessed images were used to train a U-Net model for 3-class semantic segmentation (Fig. 1B). Extensive data augmentation techniques were applied during training to enhance model robustness and generalization (Fig. S2a). This pseudo-label to deep learning approach enables high-accuracy segmentation from limited initial annotations, following a progressive enhancement strategy [36]. The trained U-Net model achieved excellent performance with a Dice coefficient of 0.976, demonstrating high fidelity in pixel classification. Compared to the initial RF pseudo-labels (Fig. 1A and Fig. S3), the U-Net segmentation exhibits superior global consistency and boundary definition. The explicit identification of edge pixels represents an improvement over traditional binary segmentation approaches [28], providing cleaner contours essential for accurate downstream instance segmentation. A representative output slice illustrating the precise delineation of particle interiors (blue), edges (yellow), and pores (red) is shown in Fig. 1B.

Based on the high-fidelity U-Net segmentation masks, instance-level segmentation was performed using a marker-controlled watershed algorithm to separate touching particles. The effectiveness of this process is illustrated in Fig. 1C and Fig. S4, where distinct colors represent individual particle instances. Zoomed-in views (Fig. 1C (i) to (iii)) demonstrate successful boundary delineation across diverse challenging morphologies commonly found in lunar regolith, including multimineral grains with varying contrast, particles with internal porosity, fine particles, and aggregates with intricate boundary geometries. However, the watershed algorithm has inherent limitations, occasionally leading to oversegmentation in brecciated particles with internal porosity (purple circle in Fig. S4g). To assess this, manual verification of the representative subregions (Fig. 1C (i) to (iii)) identified 17 errors out of 213 segmented particles, yielding an accuracy of ~92% that aligns with recent benchmarks [27]. Future implementations could benefit from incorporating shape priors or boundary confidence measures to optimize watershed seed generation strategies. A high-resolution figure (Data File S1) comparing the raw micro-CT slice with the semantic and instance segmentation is provided to illustrate the segmentation quality in detail. While local verification confirms high accuracy, global quantitative evaluation of instance segmentation accuracy for such a large and complex dataset presents substantial challenges due to the extensive effort required for generating instance-level ground truth for the 3D volume. Therefore, an indirect validation strategy was also adopted, comparing key physical metrics derived from the segmentation against trusted, independent backscattered electron (BSE) imaging analysis.

Finally, the stack of 2D instance-segmented slices was processed to reconstruct the 3D geometry of each particle using the marching cubes algorithm. A filtering protocol was applied to the initial set of reconstructed objects to ensure the data quality of the final dataset. Specifically, incomplete particles intersecting the sample volume boundary and noise artifacts smaller than a 27-voxel volume threshold were removed. This process yielded a final dataset comprising 349,740 valid, complete particles for subsequent morphological analysis, with their collective 3D rendering shown in Fig. 1D.

### Three-dimensional morphological characteristics of CE-6 lunar regolith

#### The grain size distribution and particle classification

**Fig. 2.**
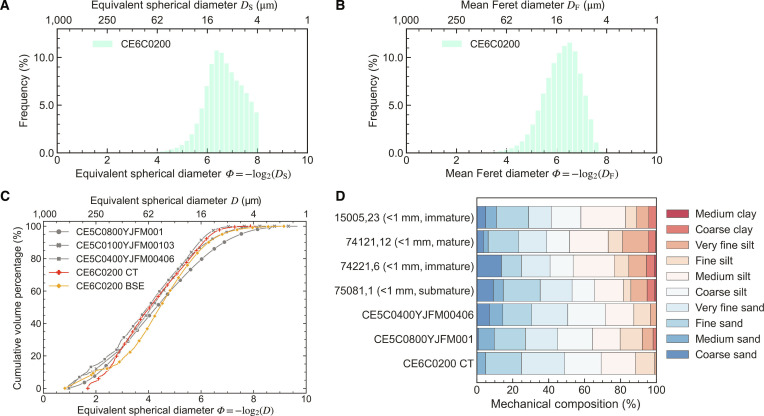
Grain size characteristics and mechanical composition of the CE-6 lunar regolith. (A) Frequency distribution of equivalent spherical diameter (*D*_S_). (B) Frequency distribution of mean Feret diameter (*D*_F_). (C) Cumulative grain size distribution of the CE-6 samples measured by micro-CT (this study; red line) and backscattered electron (BSE) (this study; orange line), compared with published data for Chang’e-5 (CE-5) samples (gray lines). (D) Volume-based mechanical composition of the CE-6 sample compared with CE-5 and selected Apollo samples, classified by *D*_S_ and maturity.

The frequency distributions of the equivalent spherical diameter (*ESD*, *D*_S_) and mean Feret diameter (*MFD*, *D*_F_) for the CE-6 lunar regolith sample are presented in Fig. 2A and B. A detailed statistical summary of these distributions is provided in Table S1. The mean *D*_S_ (11.62 μm) is smaller than the mean *D*_F_ (15.98 μm), reflecting their different definitions: *D*_S_ is calculated from particle volume assuming spherical geometry, whereas *D*_F_ is derived from the average of projection-based measurements across multiple orientations. Consequently, *D*_F_ better captures particle irregularity and elongation, yielding larger values than *D*_S_ for irregular particles.

A comparative analysis of the grain size distribution (GSD) curves for CE-5 and CE-6 lunar regolith samples is displayed in Fig. 2C. The GSD curve for the CE-6 sample (CE6C0200), obtained via micro-CT, exhibits a steeper gradient compared to the CE-5 GSD curves (CE5C0100YJFM00103 [27] and CE5C0400YJFM00406 [28]) acquired using the same technique. This indicates that the CE-6 sample has a narrower particle size range and a higher concentration of grains within that range, suggesting a more uniform composition with fewer coarse particles. Methodological variations are also evident in the comparison: the GSD curve from optical microscopy (CE5C0800YJFM001 [12]) is flatter, likely due to limitations in imaging resolution and accuracy for fine particles. When our micro-CT results are compared with our independent BSE analysis (Fig. S5), differences emerge. In the fine-particle range (<30 μm), the CT-derived GSD curve lies above the BSE-derived curve (Fig. 2C), indicating that micro-CT analysis identifies a lower proportion of the finest particles. This observation is consistent with the inherent limitations of micro-CT analysis, particularly the 27-voxel threshold that excludes the finest grains. Importantly, the comparison does not show excessive fine particle counts that would indicate widespread oversegmentation. Therefore, despite methodological differences, the comparison provides supporting evidence for the overall reliability of CT-derived GSD measurements. In the coarse-particle range, the BSE-derived curve shows greater variability due to the influence of random cross-sections on the apparent dimensions of large, irregular particles, highlighting the advantages of 3D micro-CT imaging analysis.

Quantitative analysis of the CE-6 GSD curve reveals a *d*_50_ of 60.51 μm, which is comparable to that of CE-5 regolith but considerably smaller than the ~72 μm reported for typical Apollo samples [23]. Direct comparison is complex due to methodological differences, and different techniques can yield varied results even on the same sample, as described in Introduction. Nevertheless, given that the accuracy of our GSD characterization has been established through our multilayered validation system (including robust segmentation pipeline and independent BSE cross-verification), the observed difference likely reflects a genuine distinction in the regolith properties, pointing to higher maturity for the CE-6 regolith, consistent with Li et al. [1]. Geotechnical parameters further support this interpretation. The CE-6 sample has a coefficient of uniformity *C*_u_ of 5.54 and a coefficient of curvature *C*_c_ of 0.76, both lower than the corresponding values for the CE-5 sample (*C*_u_ = 6.63 and *C*_c_ = 0.91) [28] and Apollo sample (*C*_u_ = 16.00 and *C*_c_ = 1.20) [23]. The lower *C*_u_ value confirms a less dispersed, more concentrated GSD. The *C*_c_ value of 0.76, falling outside the typical range of 1 to 3 for well-graded soils, indicates a relative deficiency in intermediate-sized particles, corresponding to the steepness of the GSD curve. These characteristics suggest that while the CE-6 soil is fine enough to fill pore spaces, its tendency toward a uniform, poorly graded structure may result in a lower bulk density compared to that of well-graded soils.

The mechanical composition of Apollo, CE-5, and CE-6 soils, classified based on the Wentworth (1922) scale [37] for clastic sediments, is illustrated in Fig. 2D. The CE-6 sample exhibits a highly concentrated composition, contrasting with the broadly distributed Apollo samples and the moderately concentrated CE-5 samples. The CE-6 soil is dominated by silt and fine sand fractions, with very fine silt (0.98%), fine silt (10.57%), medium silt (19.27%), and coarse silt (20.30%) collectively constituting over 51% of the sample. Very fine sand (24.13%) and fine sand (19.87%) represent the next most abundant components, while medium sand (4.87%) is a minor component, and coarse and medium clay fractions are negligible. This compositional profile is consistent with the steep GSD curve in Fig. 2C and supports the conclusion of a size-concentrated regolith. The enrichment in medium-to-fine grain sizes may be related to the geological context of the sampling site within the SPA basin, where different impact and weathering histories could influence GSD compared to near-side locations.

#### The particle shape characteristics

The irregular morphology of lunar regolith particles promotes interlocking, a characteristic that influences the material’s macroscopic mechanical behavior. *SPH*, a key parameter quantifying how closely a particle resembles a sphere, reflects both surface irregularity and principal axis uniformity.

**Fig. 3.**
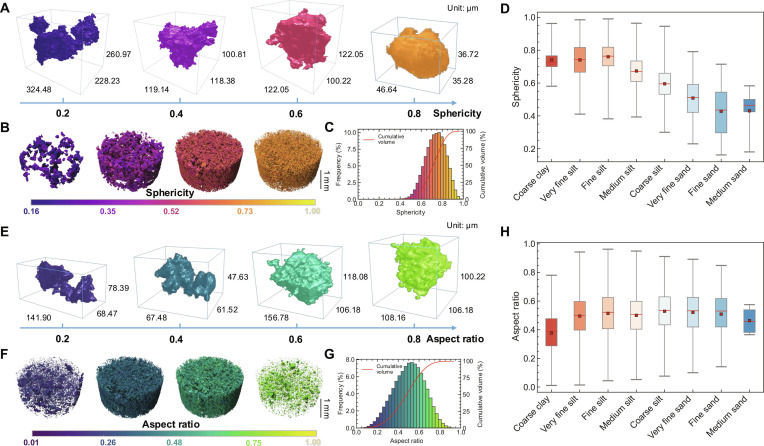
Three-dimensional morphological analysis of the CE-6 lunar regolith. (A to D) Analysis of particle sphericity. (A) 3D renderings of representative particles across a range of sphericity values. (B) Spatial distribution of sphericity within a sample subvolume, color coded by value. (C) Frequency and cumulative distributions of sphericity for the bulk sample. (D) Box plot illustrating the inverse correlation between sphericity and grain size. (E to H) Analysis of particle aspect ratio. (E) 3D renderings of representative particles across a range of aspect ratio values. (F) Spatial distribution of aspect ratio. (G) Frequency and cumulative distributions of aspect ratio. (H) Box plot showing the distribution of aspect ratio as a function of grain size.

Representative particles from the CE-6 sample with sphericity values ranging from 0.2 to 0.8 and physical sizes of approximately 30 to 300 μm are presented in Fig. 3A. Particles with high *SPH* display more uniform principal axis lengths, closely resembling spheres. The spatial and frequency distributions of *SPH* are detailed in Fig. 3B and C, with the corresponding statistical values summarized in Table S1. The frequency distribution is right-skewed, with a broad peak centered near 0.75, which may reflect the long-term effects of space weathering, where irregular, low-sphericity particles are progressively comminuted into smaller, more regular, higher-sphericity grains. The mean sphericity of 0.74 is notably lower than that of the CE-5 regolith (0.87) [28], suggesting that the CE-6 particles are, on average, more irregular.

The relationship between particle size and sphericity is quantified in Fig. 3D, revealing a clear inverse correlation whereby sphericity increases with decreasing particle size. Mean sphericity values for each size class are summarized in Table S2. This trend supports the conclusion from GSD analysis that regolith maturity increases with decreasing grain size. This size–sphericity relationship can be attributed to the presence of glassy components, which constitute an average of 29.4% of the CE-6 soil [1]. The formation of high-sphericity glass beads, particularly those under 10 μm, results from rapid cooling of molten droplets produced during meteoroid impacts [10]. This process enhances the regularity of the finest particle fractions, providing a physical basis for the observed correlation between size, shape, and maturity. The observed trend, where sphericity increases for finer particles but does not approach unity, reflects the distinct nature of lunar space weathering. Unlike terrestrial processes where abrasion by wind and water leads to rounding, lunar weathering is dominated by micrometeorite impact comminution, which reduces size without efficient rounding [27,38,39]. This explains why even mature, fine-grained lunar regolith retains considerable irregularity.

*AR* is another fundamental parameter for describing particle shape. Values approaching 1.0 indicate equant particles, while smaller values signify more elongated shapes. Figure 3E illustrates typical CE-6 particles with *AR* between 0.2 and 0.8. The spatial and frequency distributions of *AR* are shown in Fig. 3F and G, with corresponding statistical values summarized in Table S1, which are consistent with typical values of 0.4 to 0.7 reported for Apollo regolith [40]. However, the mean *AR* (0.50) is lower than that of the CE-5 regolith (0.55) [28], indicating that the CE-6 particles are generally less elongated and closer to ellipsoidal or slightly oblate shapes.

The distribution of *AR* across grain size classes is plotted in Fig. 3H, with corresponding detailed values provided in Table S2. Unlike *SPH*, *AR* shows no clear trend with particle size, remaining relatively constant across all fractions. This observation, also noted by Zhou et al. [28], suggests that particle elongation is independent of particle size. Visual comparison of Fig. 3A and E reveals that while increased *SPH* is associated with visibly smoother particle surfaces, increased *AR* is not. This distinction highlights that *AR* primarily captures geometric elongation, whereas *SPH* provides a more comprehensive description of particle form by integrating both axial proportions and surface complexity.

#### The particle overall surface roughness

The overall surface roughness of lunar regolith particles is a critical factor that influences macroscopic mechanical responses such as shear strength and bearing capacity. To quantify this characteristic, a total roughness proxy (*TRP*) was employed; this metric captures the overall undulation of the particle’s surface profile, providing a geometric measure of surface roughness.

**Fig. 4.**
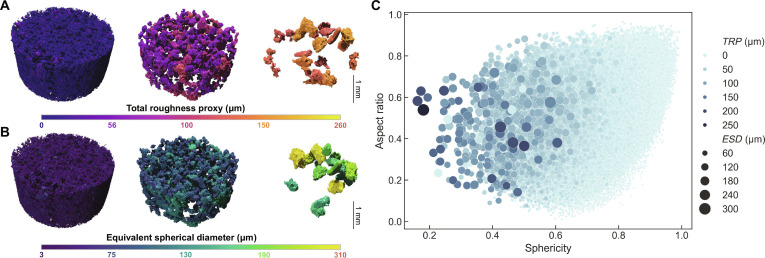
Analysis of particle roughness and its correlation with size and shape in the CE-6 lunar regolith. (A and B) 3D renderings of the particle collective within a subvolume, color coded by total roughness proxy (*TRP*) (A) and equivalent spherical diameter (*ESD*) (B) to illustrate their spatial correlation. (C) Scatter plot revealing the 4-dimensional relationship between sphericity (*x*-axis), aspect ratio (*y*-axis), *TRP* (color scale), and *ESD* (point size).

The analysis reveals strong correlations between particle roughness, size, and sphericity. Statistical *TRP* values are summarized in Table S1. The mean *TRP* (7.47 μm) is higher than the 3.42 μm reported for CE-5 soil [28], indicating that the CE-6 particles possess a rougher surface texture. As illustrated in Fig. 4A and B, the spatial distribution and magnitude of *TRP* align closely with *ESD*, suggesting that larger particles tend to be rougher. This multiparameter relationship is illustrated in the scatter plot in Fig. 4C, which maps *TRP* (color), *ESD* (point size), aspect ratio (*y*-axis), and sphericity (*x*-axis). A clear trend emerges: As sphericity increases, both *TRP* and *ESD* decrease. This finding indicates that the most spherical particles are also the smallest and smoothest, reinforcing the established conclusion that a smaller grain size correlates with higher geologic maturity. Conversely, particles with low sphericity are predominantly large and exhibit high roughness. In contrast, no systematic correlation is observed between aspect ratio and either roughness (*TRP*) or size (*ESD*), consistent with conclusions from the shape analysis. The uniform vertical distribution of point colors and sizes in Fig. 4C confirms that particle elongation is decoupled from its surface roughness and overall scale.

### Mechanical property prediction of CE-6 lunar regolith based on DEM

Based on the comprehensive morphological characterization presented above, 3D DEM simulations were conducted to investigate the macroscopic and microscopic mechanical behavior of the CE-6 lunar regolith. The CE-6 particles exhibit lower sphericity and more irregular surface morphology compared to Apollo and CE-5 samples, requiring examination of how these geometric characteristics influence bulk mechanical response. Given that sphericity was demonstrated to be a more reliable descriptor of particle geometry than aspect ratio, as detailed in “The particle shape characteristics” section, sphericity was adopted as the primary shape control parameter in the DEM model. The simulations incorporated lunar-specific low confining pressures and interparticle adhesive forces to represent the stress state and contact mechanics of near-surface lunar regolith.

#### Macroscopic analysis of DEM simulation results

**Fig. 5.**
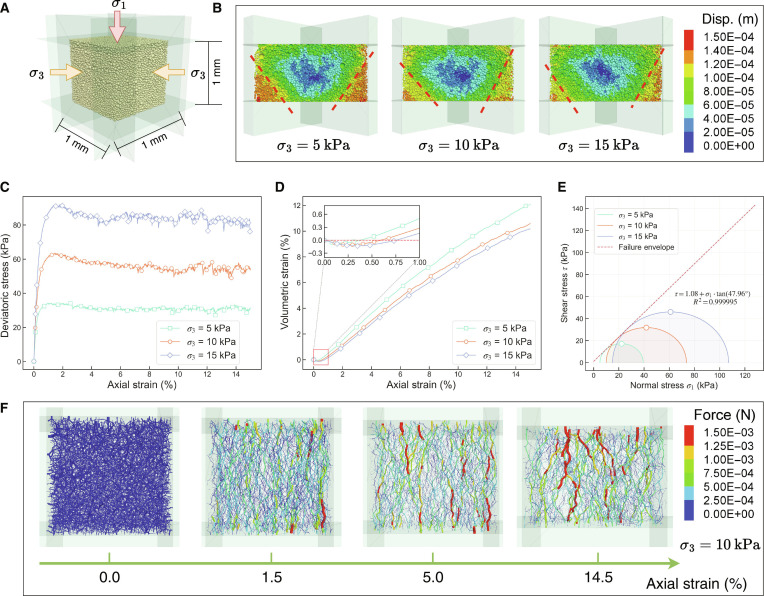
Three-dimensional discrete element method (DEM) triaxial compression simulations of CE-6 lunar regolith under low confining pressures. (A) Numerical model configuration. (B) Displacement fields at 14.5% axial strain under 3 confining pressures (*σ*_3_ = 5, 10, and 15 kPa), illustrating the development of characteristic X-shaped shear bands. (C) Deviatoric stress–axial strain curves demonstrating strain-softening behavior with peak stresses of 34.32, 63.61, and 92.04 kPa for the 3 confining pressures. (D) Volumetric strain–axial strain relationships showing initial slight compression (~0.2%, inset) followed by sustained dilation, with final volumetric strains of 12.08%, 10.60%, and 10.18%. (E) Mohr–Coulomb failure envelope fitted to peak strengths, yielding a cohesion of 1.08 kPa and an internal friction angle of 47.96°. (F) Evolution of force chain networks at *σ*_3_ = 10 kPa, showing the progression from random initial distribution (0%) to organized vertical load-bearing pathways at large strains (14.5%).

The numerical triaxial compression model, illustrated in Fig. 5A, comprised 37,226 spherical particles representing a statistically representative volume element of the regolith microstructure. The initial porosity was set to 0.46 [5], corresponding to an average relative density of approximately 83% for the 0- to 60-cm depth range [41], characteristic of dense silty soil. The displacement fields at 14.5% axial strain under 3 confining pressures (*σ*_3_ = 5, 10, and 15 kPa) are presented in Fig. 5B. The results reveal the formation of distinct shear bands exhibiting the characteristic X-shaped localization pattern [42,43]. This pattern represents a classic failure mode for dense materials in triaxial compression, and its reproduction validates the physical realism of the DEM model. The shear localization is driven by 2 coupled mechanisms. First, the strong geometric interlocking, resulting from the irregular particle shapes of the CE-6 sample, restricts particle rotation and forces strain to concentrate. Second, the sample’s high initial relative density (~83%) requires localized dilatancy (volumetric expansion) to accommodate shear, a process that further promotes band formation.

The deviatoric stress–axial strain relationships (Fig. 5C) demonstrate strain-softening behavior across all confining pressures. The stress increases rapidly during initial loading, reaching peak values at approximately 1.5% strain before gradually declining to stable residual levels. Peak deviatoric stresses for *σ*_3_ = 5, 10, and 15 kPa are 34.32, 63.61, and 92.04 kPa, respectively, with corresponding peak strains of 1.60%, 1.46%, and 1.81%. These results indicate that higher confining pressures enhance material strength and increase the magnitude of post-peak stress degradation, reflecting greater structural damage. The volumetric strain–axial strain relationships (Fig. 5D) reveal initial slight compression (approximately 0.2% as shown in the inset), followed by sustained dilation. Final volumetric strains are 12.08%, 10.60%, and 10.18% for the 3 confining pressures, respectively, exhibiting the typical dilatant behavior of dense granular materials [15,43]. The initial compression phase occurs because higher confining pressures suppress fabric expansion, promoting more compact particle arrangements. Subsequently, particle rolling and sliding induce irreversible volumetric expansion, with higher confining pressures providing greater constraint on dilatancy, resulting in reduced final volumetric strains.

The Mohr–Coulomb failure envelope fitted to the peak strengths (Fig. 5E) yields a cohesion *c* of 1.08 kPa and an internal friction angle *φ* of 47.96°. These values are high compared to data from previous lunar missions. Geotechnical estimates derived from Surveyor 3 and 7 lander data suggested *c* = 0.35 to 0.7 kPa and *φ* = 35° to 37° [24], while parameters inferred from Apollo mission analyses (e.g., soil penetration tests and rover track observations) typically fell within *c* = 0.1 to 1.0 kPa and *φ* = 30° to 50° [25]. Although samples from Soviet Luna 16 and 20 missions exhibited higher cohesion (*c* = 3.9 to 5.9 kPa) under high confining pressures, their reported friction angles were markedly lower (*φ* = 20° to 25°) [5], and the experimental conditions were not directly comparable to the lunar surface environment.

The enhanced mechanical properties of CE-6 regolith can be attributed to its unique geological context and composition. The sample originates from the southern rim of the Apollo crater within the SPA basin, a region with complex history of material excavation and mixing [1,44]. Compositional analysis of the returned samples indicates that the sample is a mixture of materials from distinct provenances: 93.3% local basalts and 6.1% materials sourced from the wider SPA basin [45]. The disparity in particle size and morphology between these components promotes the formation of a complex and effective mechanical interlocking network. The pronounced geometry interlocking of CE-6 particles is quantitatively captured by their low mean 3D sphericity (*SPH* = 0.74) measured in this study. This value is smaller than the mean *SPH* of 0.87 reported for near-side CE-5 regolith, which was analyzed using the same 3D micro-CT technique [28].

In DEM simulations, this statistical finding was used to calibrate a high rolling friction coefficient, effectively simulating the strong resistance to particle rotation caused by irregular shapes. Such structural interlocking enhances resistance to shear slippage, resulting in a high internal friction angle. Furthermore, the substantial cohesion is linked to the existence of glassy agglutinates (~30%), which exceeds that of the CE-5 regolith (~21%) [1,21]. Although the morphological (rolling friction, *μ*_r_) and adhesive (surface energy, *γ*) effects are inherently coupled, the sensitivity analysis clearly delineates their primary controls: *μ*_r_—as a morphological proxy—predominantly controls the internal friction angle (*φ*), whereas *γ* primarily controls cohesion (*c*) (Fig. S6). The high shear strength of the CE-6 regolith is therefore attributed to this morphology-driven particle interlocking, likely augmented by cementation effects from the substantial glassy agglutinate phase formed by micrometeorite impact melting [46–48]. This provides an empirical basis for future lunar engineering, construction, and resource utilization studies.

The evolution of force chain networks for *σ*_3_ = 10 kPa is exemplified in Fig. 5F. Initially (0% strain), force chains are randomly distributed with no preferred orientation. At peak stress (1.5% strain), strong vertical force chains begin to develop, forming the structural backbone that spans the emerging shear band. With continued deformation (5% to 14.5% strain), these vertical force chains intensify and undergo branching and reorganization, while weaker chains rupture and disappear, leading to distinct load-bearing pathways. This process represents the fundamental micromechanical evolution of lunar regolith under shear, where frictional sliding and local interlocking continuously redistribute stresses. The evolution highlights a structure-dominated redistribution of stress, a defining feature of granular materials at low confining pressures.

#### Micromechanical mechanism analysis

To elucidate the micromechanical mechanisms governing CE-6 lunar regolith behavior during triaxial compression, a quantitative analysis of the DEM simulation results is presented from a microstructural evolution perspective. The analysis focuses on 3 key aspects: the evolution of particle contact networks, contact failure modes, and the development of anisotropy patterns, aiming to reveal the microscopic basis underlying macroscopic mechanical response.

**Fig. 6.**
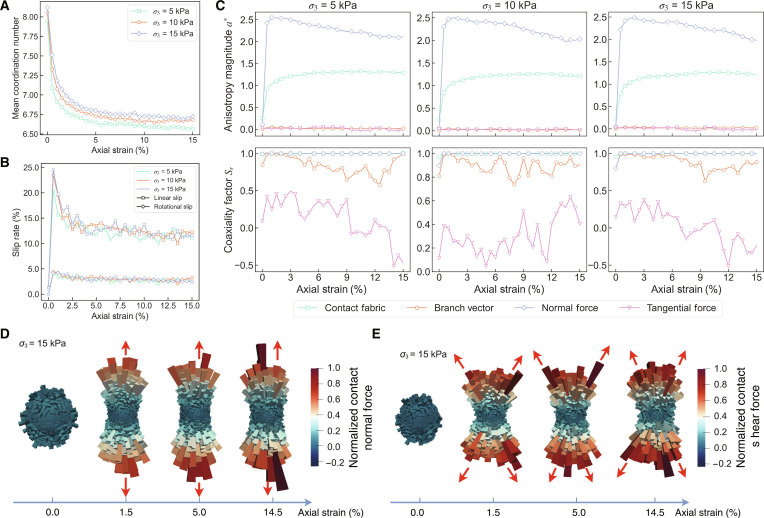
Micromechanical analysis of CE-6 lunar regolith during triaxial compression. (A) Evolution of mean coordination number with axial strain under 3 confining pressures, showing rapid decrease below 5% strain followed by stabilization at ~6.6. (B) Distribution of contact failure types, demonstrating the dominance of linear slip over rotational slip due to enhanced geometric interlocking of irregular particles. (C) Development of anisotropy magnitude (top) and coaxiality factors (bottom) for contact fabric, branch vector, normal force, and tangential force components across the 3 confining pressures. Normal force anisotropy peaks at ~2.5 at 1.5% strain, while contact fabric anisotropy stabilizes at ~1.3 after 5% strain. (D and E) 3D spatial distributions of normalized normal forces (D) and tangential forces (E) at *σ*_3_ = 15 kPa, illustrating the transition from initial isotropic distribution (0% strain) to highly organized anisotropic patterns (14.5% strain), with normal forces concentrating vertically and tangential forces forming an X-shaped pattern consistent with macroscopic shear band development.

The evolution of mean coordination number under different confining pressures is presented in Fig. 6A. The coordination number decreases rapidly for axial strains below 5%, subsequently stabilizing at approximately 6.6, with higher confining pressures yielding higher coordination numbers. This reflects contact network reorganization during shearing: initially, the model contains numerous randomly distributed contact points, but as deviatoric stress increases, lateral contacts rupture, while vertical contacts that bear the primary shear stress strengthen [49,50]. This evolution parallels the force chain development observed in Fig. 5F. Notably, the strain at which the coordination number stabilizes (~5%) coincides with the strain at which the macroscopic mechanical behavior transitions to a residual state, indicating that micromechanical contact structure stabilization forms the microscopic foundation for macroscopic mechanical equilibrium.

The distribution characteristics of contact failure types under different confining pressures are revealed in Fig. 6B. The results demonstrate that contact failure in the model is predominantly governed by linear slip, with rotational slip accounting for a relatively small proportion. This phenomenon can be attributed to the low sphericity and highly irregular shape characteristics of CE-6 particles, which enhance geometric interlocking effects between particles and suppress particle rolling motion; this finding is also consistent with previous DEM studies [15]. Additionally, the proportion of contact failure exhibits a rapid increase before the peak strain of 1.5% and then gradually stabilizes after strains exceed 5%, corresponding to the post-peak softening phase of the macroscopic stress–strain curves.

The evolution of anisotropy magnitude in the model is illustrated in Fig. 6C. The development patterns are consistent across different confining pressures. Normal force anisotropy is the most pronounced, with its magnitude parameter *a*^n^ reaching a maximum value of approximately 2.5 at a peak strain of 1.5%. Contact fabric anisotropy *a*^c^ ranks second, rapidly growing to approximately 1.0 before the peak and then slowly increasing to a stable value of 1.3 at 5% strain. In contrast, branch vector and tangential force anisotropies are relatively weak, with *a*^d^ and *a*^t^ both approaching zero. Coaxiality coefficient analysis reveals that $S_{r}^{n}$ and $S_{r}^{c}$ are nearly unity, indicating that normal forces and contact fabric are highly aligned with the principal stress direction and constitute the primary contributors to anisotropy development. $S_{r}^{d}$ values are generally greater than 0.8, suggesting that branch vectors are also reasonably aligned with the principal stress direction, albeit with a low magnitude. $S_{r}^{t}$ exhibits the smallest values with notable dynamic fluctuations, indicating that tangential forces fail to establish stable preferential orientation.

Comprehensive analysis of these micromechanical trends reveals the underlying physics of regolith’s behavior. The development of normal force anisotropy is consistent with the trends in contact failure and macroscopic deviatoric stress, all peaking at 1.5% strain. This indicates that the material’s peak strength is governed by the formation of a strong, anisotropic network of normal force chains that collapses once its load-bearing capacity is exceeded. In contrast, the consistently low tangential force anisotropy suggests that while local sliding friction is active, the overall shear strength is dominated by geometric interlocking of irregular particles (simulated by rolling friction), which inhibits the formation of directionally aligned sliding failure mechanisms.

Conversely, the development of geometric contact fabric anisotropy aligns with the evolution of the coordination number, reaching a stable state at approximately 5% strain. This stabilization of coordination number serves as a micromechanical criterion for the onset of the macroscopic residual state, as it signifies that the particle contact network has reached dynamic equilibrium. This differential development, where the force fabric peaks early while geometric fabric evolves more slowly, reflects the coupled evolution of mechanical and geometric structures in dense granular materials during shearing.

The spatial distribution evolution of normalized normal and tangential forces in 3D space with *σ*_3_ = 15 kPa is exemplified in Fig. 6D and E. Initially, at 0% strain, both force components exhibit uniform isotropic distributions. As shearing progresses, normal forces gradually concentrate in the vertical direction, while tangential forces redistribute along the principal stress direction, forming a distinct X-shaped pattern (indicated by red arrows). This spatial distribution characteristic is consistent with the shear band morphology revealed by the particle displacement field in Fig. 5B, demonstrating the connection between macroscopic shear localization and microscopic force chain reorganization.

In summary, the micromechanical analysis reveals the contact network reconstruction mechanism of CE-6 lunar regolith during triaxial compression: transitioning from an initial isotropic random contact state, through contact failure and reorganization, to ultimately form a highly anisotropic load-bearing network. This microscopic evolutionary process provides crucial microscopic insights for understanding the physical essence of lunar regolith macroscopic mechanical behavior and establishes a theoretical foundation for developing micromechanics-based constitutive models for lunar regolith.

## Conclusion

This study provides comprehensive characterization of CE-6 far-side regolith through an integrated pipeline that combines high-resolution micro-CT imaging with a semisupervised iterative training framework. This approach enabled robust analysis of 349,740 individual particles, revealing that CE-6 grains are more irregular and exhibit a narrower GSD with a reduced coarse particle content compared to near-side materials. A median diameter of 60.51 μm and a mean 3D sphericity of 0.74 define a distinct physical state that reflects the geological history of the lunar far side. Particle aspect ratio and roughness analyses further characterize the morphological properties of these far-side materials.

To investigate how these morphologies govern mechanical behavior, shape-informed DEM simulations were conducted under relevant lunar conditions of 5- to 15-kPa confinement. The results show that far-side regolith is intrinsically stronger and more cohesive than its near-side counterparts. A derived internal friction angle of 47.96° and a cohesion of 1.08 kPa exceed Surveyor estimates and lie at the upper bound of Apollo data. This enhanced mechanical performance is attributed primarily to geometric interlocking resulting from high particle irregularity. This mechanism, which may be further augmented by cementation from a substantial glassy agglutinate phase, is the direct consequence of the morphological characteristics quantified in this study. In parallel, the simulations successfully reproduced the formation of characteristic X-shaped shear bands, a failure signature consistent with granular material theory.

This modeling approach necessitates key simplifications due to the lack of direct calibration data, including estimating rolling resistance (*μ*_r_) from sphericity via an extrapolated empirical formula [15,51] and simplifying adhesion using literature-based surface energy [52,53]. The results should thus be viewed as morphology-based benchmark predictions requiring validation. Future work should integrate high-fidelity morphology (e.g., via clump/polyhedral elements) and roughness effects more directly into DEM, preferably calibrated against mechanical tests.

Nevertheless, by establishing these benchmark geotechnical parameters, this work provides a foundation for engineering design and in situ resource utilization on the lunar surface. Furthermore, while this study does not provide conclusive insights into the specific geological processes responsible for these morphologies, it provides the first large-scale 3D morphological dataset for CE-6 regolith, which is an essential prerequisite for future investigations into space weathering mechanisms on the lunar far side. The integrated characterization pipeline is also broadly applicable, offering a nondestructive methodology for analyzing other extraterrestrial granular materials and advancing comparative planetology.

## Materials and Methods

A comprehensive list of abbreviations and their definitions is provided in Table S3 in the Supplementary Materials.

### The CE-6 lunar regolith sample

The lunar regolith sample used in this study, with the official designation CE6C0200, was provided by the China National Space Administration. This sample is a subsample of the 1,935.3 g of scooped material returned by the CE-6 mission from the southwest part of Apollo basin (41.625°S, 153.978°W) on the lunar far side. The regolith in this region is known to be primarily composed of basaltic clasts with minor nonbasaltic ejecta.

### Micro-CT image segmentation and reconstruction

Micro-CT analysis was performed on a high-resolution micro-CT dataset of approximately 30 mg of lunar regolith sample CE6C0200. The scan resulted in 1,000 consecutive axial slices with an isotropic voxel resolution of 0.9923 μm (full details are provided in Note S1). The morphological and structural complexity of lunar regolith particles presents challenges for segmentation. Particle sizes span multiple scales, internal features like pores and mineral inclusions create variable contrast, and irregular boundaries are common in brecciated fragments (Fig. S7). To capture these complex structures, a multistage workflow was developed, beginning with image preprocessing and feature extraction, followed by a self-training pipeline for semantic segmentation, and concluding with instance segmentation, 3D model reconstruction, and systematic filtering to preclude artifacts and ensure the data quality of the final particle dataset. A key innovation in this process is the use of a 3-class segmentation scheme (particle, pore, and edge) to provide essential support for subsequent instance-level segmentation, as detailed in the following sections.

#### Iterative self-training framework for pseudo-label generation

The segmentation process began with preprocessing the raw 2D micro-CT slices to enhance quality. This involved contrast improvement using the contrast-limited adaptive histogram equalization algorithm, followed by noise reduction using a nonlocal means filter selected for its edge-preserving properties (Fig. S1a). Subsequently, a multidimensional feature vector was constructed for each pixel to provide comprehensive information for classification (Fig. S1b), encompassing grayscale intensity, edge features (difference of Gaussian and Gaussian gradient magnitude), and texture features (histogram of oriented gradient and statistical texture extraction).

To address the challenges of large dataset size and annotation subjectivity, an iterative self-training framework was employed to generate pseudo-labels from sparse manual annotations [54]. Initially, a small, representative set of pixels across key regions were manually labeled into 3 classes: “edge”, “particle”, and “pore”. These sparse labels, combined with the extracted multidimensional features, were used to train 3 machine learning classifiers: linear discriminant analysis [55], decision tree [56], and RF. The performance of these classifiers was evaluated (Fig. S1c). The RF model, configured with 100 estimators (implemented using scikit-learn), demonstrated superior robustness in maintaining boundary continuity and resolving local complexities, such as intraparticle pores and regions with varying contrast. Based on this comparative evaluation, the RF model was selected to generate the final 3-class pseudo-label dataset across the relevant image slices. This dataset served as training input for the subsequent deep-learning-based semantic segmentation stage.

#### Semantic segmentation based on U-Net

The final semantic segmentation was performed using a U-Net convolutional neural network trained on the 3-class RF-generated pseudo-labels. To enhance model performance, the training dataset was expanded via data augmentation, as illustrated in Fig. S2a (specific parameters are detailed in Note S2). The techniques included random horizontal and vertical flipping, rotation, scaling, brightness adjustments, and the addition of Gaussian noise. The images were subsequently divided into patches, creating a final dataset of 82,621 training and 496 validation patches.

The U-Net model utilized a standard encoder–decoder architecture (Fig. S2b). The model was trained with the Adadelta optimizer and a weighted categorical cross-entropy loss function, which emphasized the edge class to improve boundary detection. Training was halted at 131 epochs by an early stopping mechanism to mitigate overfitting. (Full architectural details and training hyperparameters are provided in Note S3.)

#### Particle instance segmentation, reconstruction, and filtering

The pixel-level classification masks from the U-Net model were used to generate instance-level segmentation, separating each individual particle. This was achieved using a marker-controlled watershed algorithm, with detailed steps illustrated in Fig. S4. First, the Euclidean distance transform was applied to the binary particle masks to generate a distance map, assigning each particle pixel a value corresponding to its distance from the nearest boundary (Fig. S4a and b). Next, local maxima within this distance map were identified and refined through nonmaximum suppression and thresholding to serve as discrete seed points, or markers, representing the core of each particle (Fig. S4c). Finally, the watershed transformation was performed on the inverted distance map using these markers, effectively delineating the boundaries separating all contacting particles (Fig. S4d and e).

The instance segmentation process was applied to the entire stack of 1,000 micro-CT slices, resulting in an initial set of 390,285 particle instances. To ensure the data quality of the final dataset, 2 filtering criteria were applied: (a) any particle making contact with the cylindrical boundary of the sample volume was removed to exclude incomplete fragments (red circles in Fig. S4e), and (b) any object with a volume of fewer than 27 voxels was discarded as likely noise or negligible microfragment. This threshold was chosen based on established practice in 3D micro-CT analysis, which requires a minimum of 3 voxels in each dimension for robust quantification [57]. Furthermore, a sensitivity analysis (Fig. S8) confirmed that key statistical metrics (GSD and mean *SPH* and *AR*) remain stable across volume thresholds from 27 to 300 voxels. Therefore, the 27-voxel threshold was retained to preserve the statistically representative fine fraction while ensuring methodological consistency [28]. After this filtering process, a final dataset of 349,740 valid, complete particles remained for subsequent analysis (Fig. S4f). The final step was to reconstruct the 3D surface mesh for each particle using the marching cubes algorithm, creating a 3D digital representation of the regolith sample.

### BSE analysis for validation

After the micro-CT scan, a portion of the same CE6C0200 sample was used for BSE analysis, which was conducted using scanning electron microscopy at 15 kV with a beam current of 10 nA on a 2.54-cm-diameter circular target. Approximately 1.52 million particles were reconstructed, with particles smaller than 4 pixels and boundary-contacting particles being excluded from size distribution analysis (full details are provided in Note S4). The objective of this analysis was to provide independent validation of the GSD obtained from the 3D micro-CT segmentation.

### Definition and calculation of morphological parameters

Following 3D reconstruction of individual particles, 7 characteristic parameters were calculated for each of the 349,740 valid particles (Table S4). These parameters were categorized into 2 classes: geometric (G class) and shape (S class).

The G-class parameters quantify the particles’ fundamental size and dimensions. Particle volume (*VOL*) was determined as the total volume occupied by its constituent labeled voxels. No pore-filling operations were performed to preserve the true physical volume of particles containing natural internal voids, such as agglutinates and breccias. Surface area (*SA*) was estimated using the weighted local configurations method, which sums the area contributions from local 2 × 2 × 2 voxel patterns, providing robust measurement for small particles [58]. From the volume, *ESD*, representing the diameter of a volume-equivalent sphere, was derived. Additional dimensional metrics included *MFD*, defined as the average of the minimum and maximum caliper distances measured over multiple orientations.

The S-class parameters describe particle form and surface texture. Sphericity quantifies the particle’s resemblance to a perfect sphere and was calculated as $\mathrm{SPH}=\sqrt[{3}]{36\pi \mathrm{VO}L^{2}}/\mathrm{SA}$ [22]. *AR* is defined as the ratio of the particle’s shortest principal axis *L*_S_ to its longest principal axis *L*_L_ [29]. Global surface texture was estimated using *TRP*, which is the average difference between the maximum radius *R*_max_ and the minimum radius *R*_min_ from the projected centroid to its contour across 3 orthogonal planes.

All particles were classified into size classes for comparative analysis (e.g., in Figs. 2D and 3D). The classification is based on the standard Wentworth (1922) grain size scale [37], which uses a geometric progression with a constant ratio of 2 between class boundaries. For example, the sand class is subdivided into very fine sand (1/16 to 1/8 mm), fine sand (1/8 to 1/4 mm), medium sand (1/4 to 1/2 mm), coarse sand (1/2 to 1 mm), and very coarse sand (1 to 2 mm). All classifications in this study are based on the particle’s *ESD*.

### Mechanical simulation

To investigate the macroscopic mechanical behavior and its connection to the observed particle morphology, 3D DEM simulations were conducted using the PFC3D 6.0 software platform.

#### DEM model setup

The DEM simulations employ an approach where geometrically simple spheres represent particles, while the complex effects of the real particles’ irregular shapes and surface adhesion are captured through an enhanced contact model. To simulate the behavior of fine lunar regolith particles in a vacuum environment, the Johnson–Kendall–Roberts cohesive contact model was employed [59–61]. This model extends the standard Hertz–Mindlin theory to account for van der Waals forces, which are notable for lunar regolith particles and manifest as interparticle adhesion [52,62]. The Johnson–Kendall–Roberts model calculates normal force and tangential force between 2 contacting particles, accounting for both elastic Hertzian forces and adhesive van der Waals forces. To distinctly capture the mechanics of nonspherical particles, the model was augmented with a rolling friction model that applies a resisting bending moment (detailed formulations for the contact model are provided in Note S5). The simulation parameters are listed in Table 1.

**Table 1.** DEM simulation parameters adopted in this study

Parameter name	Parameter value	References
Particle density *ρ* (kg·m^−3^)	3,195	[12]
Surface energy density *γ* (J·m^2^)	0.01	[52,53]
Effective modulus E^*^ (GPa)	4.49	[72]
Poisson’s ratio *ν*	0.4	[73]
Shear modulus *G* (GPa)	1.604 ^a^	-
Interparticle friction *μ*_p_	0.76	[72]
Wall–particle friction *μ*_w_	0.0	-
Rolling friction *μ*_r_	2.934 ^b^	[51]
Damping ratio *α*	0.7	[74]

^a^ Shear modulus *G* is calculated as *G* = 0.5E^*^/(1 + *ν*).

^b^ Rolling friction *μ*_r_ is calculated as [51] *μ*_r_ = 0.1963*S*^−8.982^, where *S* is the mean 3D sphericity of CE-6 lunar regolith, i.e., 0.74 in this study.

Directly modeling the actual GSD of lunar regolith in DEM is computationally challenging due to the vast number of fine particles [63]. Conventional approaches often involve simplifying the GSD by using artificially narrow distributions to enhance computational efficiency [15,16,43,64]. However, these methods lack rigorous theoretical basis and are not uniquely determined. To address the challenge of representing the full GSD while maintaining computational feasibility, this study employed a deterministic method similar to the unified granular configuration theory [65,66], and used the Weibull distribution to define the GSD with scale parameter *d*_s_ and shape parameter *d*_f_ (the formulation is provided in Note S5). The GSD for the numerical sample was generated by fitting this function to the experimental data of the CE-6 regolith. To ensure representativeness while maintaining computational feasibility, the *d*_50_ of the simulation was matched to the experimental value, and a shape parameter *d*_f_ of 7 was selected. This configuration ensures that the resulting GSD (Fig. S9) accurately encompasses the key control particle sizes (*d*_30_, *d*_50_, and *d*_60_) of the real sample, providing a reproducible GSD that is both representative and computationally manageable.

#### Simulation procedure and analysis

The mechanical behavior of the lunar regolith sample was investigated using a simulated drained triaxial compression test. To enhance computational efficiency, the density scaling method was employed for this quasi-static process [67]. To analyze the evolution of the sample’s internal structure, several micromechanical metrics were calculated throughout the simulation. First, the mode of contact failure was monitored, distinguishing between rotational slip and linear slip to understand the dominant failure mechanisms. Second, to characterize the density of the force-bearing contact network, the mechanical coordination number *Z*_m_, which excludes non-load-bearing particles, was calculated as [68]

$$Z_{m}=\frac{2N_{c}-N_{b1}}{N_{b}-N_{b1}-N_{b0}}$$ (1)

where *N*_c_ is the total number of contacts, *N*_b_ is the total number of particles, *N*_b1_ is the number of particles with only one contact, and *N*_b0_ is the number of particles with no contacts.

Third, to quantify the directional evolution of the sample’s geometric and mechanical structure, 4 sources of anisotropy were calculated based on the framework proposed by Oda [69] and Guo and Zhao [70]. For each source, an anisotropy tensor *a_ij_* is first computed: contact fabric anisotropy $a_{\mathrm{ij}}^{c}$ describes the directional distribution of contact normal, branch vector anisotropy $a_{\mathrm{ij}}^{d}$ describes the anisotropy of the vectors connecting the centers of contacting particles, normal force anisotropy $a_{\mathrm{ij}}^{n}$ quantifies the directional distribution of normal contact forces, and tangential force anisotropy $a_{\mathrm{ij}}^{t}$ quantifies the directional distribution of tangential contact forces. From each of these 4 tensors $a_{\mathrm{ij}}^{*}$, 2 scalar parameters are derived to provide a comprehensive measure of anisotropy: anisotropy magnitude a^*^ represents the intensity of the anisotropy, and coaxiality factor *S*_r_ is a scalar value from −1 to 1 that measures the degree of alignment between the anisotropy tensor and the deviatoric stress tensor. This results in 8 scalar parameters (*a*^c^, *a*^d^, *a*^n^, and *a*^t^ and $S_{r}^{c}$, $S_{r}^{d}$, $S_{r}^{n}$, and $S_{r}^{t}$) that fully characterize the evolution of the micromechanical anisotropy (detailed formulas for the anisotropy tensors are provided in Note S6).

## Data Availability

The data of this study are available from the corresponding authors upon request.

## Supplementary Materials

Supplementary 1 Notes S1 to S6 Figs. S1 to S9 Tables S1 to S4 Data File S1
