# Supplementary material for: Particle Morphology Controls the Bulk Mechanical Behavior of Far-Side Lunar Regolith from Chang’e-6 Samples and Deep Learning
Source: Research (Wash D C). 2026 Jan 8;9:1064. doi: 10.34133/research.1064 (PMC12779894; doi:10.34133/research.1064)
Supplement: Supplementary 1 — Notes S1 to S6 Figs. S1 to S9 Tables S1 to S4 Data File S1 [file research.1064.f1.zip › 1224_Supplementary_Materials_v1.docx]

**Table of contents**

**Note S1.** Micro-CT Imaging of CE-6 Lunar Regolith.

**Note S2.** Data Augmentation Parameters.

**Note S3.** U-Net Architecture Details.

**Note S4.** BSE Imaging of CE-6 Lunar Regolith.

**Note S5.** DEM Model Formulations.

**Note S6.** Formulation of Anisotropy Tensors.

**Figure S1.** Schematic of the iterative self-training framework for semantic segmentation of lunar regolith particles from micro-CT images.

**Figure S2.** U-Net framework and performance for multi-class semantic segmentation of lunar regolith.

**Figure S3.** Comparison of segmentation performance between RF and U-Net.

**Figure S4.** Instance segmentation of individual particles on a representative 2D slice using a marker-controlled watershed algorithm.

**Figure S5 .** Quality control in 2D particle size analysis from BSE imaging.

**Figure S6**. Parameter sensitivity analysis of DEM triaxial compression tests showing the decoupled effects of particle morphology and adhesion.

**Figure S7.** Challenges in automated segmentation of lunar regolith micro-CT images.

**Figure S8.** Sensitivity analysis of morphological statistics to variations in the particle volume filtering threshold.

**Figure S9.** Generation of the representative grain size distribution for DEM simulation.

**Table S1.** Statistical Summary of CE-6 Morphological Parameters.

**Table S2.** Mean Shape Parameters per Particle Size Class.

**Table S3.** List of Abbreviations.

**Table S4.** Seven 3D morphological characteristics of CE-6 Lunar Regolith.

**Data file S1.** High-resolution visual validation of the multi-stage segmentation pipeline on a representative micro-CT slice.

# Micro-CT Imaging of CE-6 Lunar Regolith

Approximately 30 mg of lunar regolith from sample CE6C0200 was transferred into a precision quartz tube with an inner diameter of 2.0 mm and mounted vertically to minimize preferential particle orientation. High-resolution micro-CT was performed using a FEI Heliscan MicroCT system (Thermo Fisher Scientific, USA) at the Institute of Geology and Geophysics, Chinese Academy of Sciences. The sample stage executed a helical trajectory during acquisition to ensure comprehensive angular coverage and mitigate cone-beam artifacts.

The scanning parameters were optimized for high-contrast imaging of silicate minerals: a source voltage of 80 kV, a tube current of approximately 90 μA, and no additional filtration. Several thousand projection radiographs were acquired, resulting in an isotropic voxel resolution of 0.9923 μm with a field of view of 2516 × 2516 pixels. Subsequently, 1,000 consecutive axial slices from the central, most stable region of the sample were reconstructed using a filtered back-projection algorithm with corrections for beam hardening and ring artifacts.

# Data Augmentation Parameters

To enhance the robustness of the deep learning model, the training dataset was expanded through extensive data augmentation. The following transformations and their corresponding parameter ranges were applied to the raw micro-CT image patches. First class was geometric transformations: Random horizontal flip was applied with a probability of 50%, random vertical flip was applied with a probability of 50%, random rotation angles were sampled uniformly from the range [-180°, +180°], random scaling factors were sampled uniformly from the range [0.9, 1.1]. Second class was photometric transformations: Brightness levels were adjusted by a factor sampled uniformly from the range [0.1, 1.1], gaussian noise with a mean of 0 and a standard deviation sampled uniformly from the range [0, 0.01] were added to the images.

# U-Net Architecture Details

The semantic segmentation task was performed using a standard U-Net architecture, implemented in PyTorch. The network consists of an encoder (contracting path) and a decoder (expansive path) with skip connections. The encoder is composed of five sequential blocks. Each block consists of two 3×3 convolutional layers, each followed by a Rectified Linear Unit (ReLU) activation function and batch normalization. A 2×2 max pooling operation with a stride of 2 is applied at the end of each block for downsampling. The number of feature channels doubles after each block, starting from 64 and increasing to 1024. The bottleneck connects the encoder and decoder and consists of two 3×3 convolutional layers, each followed by ReLU and batch normalization. The decoder is composed of four sequential blocks. Each block begins with a 2×2 transposed convolution for upsampling, which halves the number of feature channels. The upsampled feature map is then concatenated with the corresponding feature map from the encoder via a skip connection. This is followed by two 3×3 convolutional layers, each with ReLU activation and batch normalization. The final layer is a 1×1 convolution with a softmax activation function, which produces the final three-class (particle, pore, boundary) probability map.

The U-Net model was trained on 128 × 128 pixels patches extracted with a 25% step size, corresponding to a 32-pixel stride. The training utilized the Adadelta optimizer with a learning rate of 1.0 and an epsilon of 1×10⁻⁶, alongside a weighted Categorical Cross-Entropy loss function. To regularize the training, the validation loss was monitored, and the learning rate was automatically reduced if the validation loss showed no improvement for 10 consecutive epochs. Furthermore, the training process was configured to terminate if the validation loss failed to improve by at least 1×10⁻⁴ over a 15-epoch period. Following this criterion, the training automatically halted at 131 epochs.

# BSE Imaging of CE-6 Lunar Regolith

BSE imaging was conducted using a scanning electron microscope operated at a 15 kV accelerating voltage with a beam current of 10 nA. The analysis target consisted of a circular area with a diameter of 1 inch (2.54 cm), ensuring representative sampling of the CE-6 regolith. High-resolution BSE images were acquired at multiple overlapping fields of view to achieve complete coverage of the target area. Image processing and particle reconstruction yielded approximately 1.52 million individual particles from the BSE datasets. To ensure statistical reliability, a systematic filtering protocol was applied to exclude noise artifacts and incomplete geometries. Particles smaller than 4 pixels were removed as potential imaging noise, while boundary-contacting particles were excluded to prevent size bias from incomplete particle boundaries. This quality control procedure retained only complete particles within the field of view for accurate size distribution calculations, as illustrated in **Figure S5**  where valid particles are highlighted in color while excluded particles remain in grayscale.

# DEM Model Formulations

This section provides the specific mathematical formulations for the models used in DEM simulations.

**1. JKR Cohesive Contact Model**

The JKR model calculates the normal force (*F*_n_) and tangential force (*F*_t_) between two contacting particles. The normal force is a combination of the elastic Hertzian contact force and the adhesive force^[61]^:

$$\begin{aligned} F_{n}=\frac{4E^{*}}{3R^{*}}\delta_{n}^{3}-4\sqrt{\pi E^{*}\gamma}\delta_{n}^{\frac{3}{2}}\#\left( S1 \right) \end{aligned}$$

where *δ*_n_ is the normal overlap, *E*^*^ is the equivalent Young's Modulus, *R*^*^ is the equivalent radius, and *γ* is the surface energy density, which governs the magnitude of the adhesive force.

The tangential force is calculated based on the tangential overlap *δ*_t_ and the tangential stiffness *k*_t_^[61]^:

$$\begin{aligned} F_{t}=-k_{t}\cdot\delta_{t}\#\left( S2 \right) \end{aligned}$$

The tangential force is limited by Coulomb's law of friction, $|F_{t}|\leq\mu_{s}|F_{n}|$, where *μ*_s_ is the coefficient of static friction.

To account for resistance to particle rotation, a rolling friction model was also included. The model applies a resisting bending moment *M*_b_ to the contacting surfaces^[61]^:

$$\begin{aligned} M_{b}=-k_{b}\cdot\theta_{b}\#\left( S3 \right) \end{aligned}$$

where *k*_b_ is the bending stiffness and *θ*_b_ is the relative bending rotation. This moment is limited by a maximum value proportional to the normal force: $|M_{b}|\leq\mu_{r}R^{*}|F_{n}|$, where *μ*_r_ is the coefficient of rolling friction.

**2. Grain Size Distribution**

A Weibull distribution function was used to define the GSD. The cumulative distribution is given by:

$$\begin{aligned} F\left( d \right)=1-\exp\left[ -\left( \frac{d}{d_{s}} \right)^{d_{f}} \right]\#\left( S4 \right) \end{aligned}$$

where *d* is the particle diameter, *d*_s_ is the scale parameter, and *d*_f_ is the shape parameter.

**3. Simulation Procedure**

To enhance computational efficiency for this quasi-static process, the density scaling method was employed. This technique involves artificially increasing particle inertial mass to allow for a larger stable time step, thereby accelerating the model's convergence to a state of equilibrium. Since the simulation is quasi-static, this scaling of inertial properties does not affect the final static mechanical response.

The procedure consisted of two stages: First, the virtual sample, with an initial porosity of 0.46, was isotropically consolidated to one of three target confining pressures (*σ*₃): 5, 10, or 15 kPa. Following consolidation, axial strain was applied at a constant rate of 0.01% while the confining pressure was maintained, simulating the shearing process until a final axial strain of 15.0% was reached.

# Formulation of Anisotropy Tensors

This section details the formulas used to quantify the four sources of anisotropy in the granular assembly. Anisotropy is decomposed into two geometric and two mechanical sources^[70]^. The first one is contact fabric anisotropy *a* c *ij* which quantifies the directional preference of contact normals:

$$\begin{aligned} a_{ij}^{c}=\frac{15}{2}\Phi_{ij}^{'}\#\left( S5 \right) \end{aligned}$$

where *Φ ' ij* represents the deviatoric part of *Φ_ij_*, which is calculated as:

$$\begin{aligned} \Phi_{ij}=\frac{1}{N_{c}}\sum_{c\in N_{c}} n_{i}n_{j}\#\left( S6 \right) \end{aligned}$$

where *N*_c_ is the total number of contacts, and *n_i_* is the unit normal vector at the contact plane.

The second one is branch vector anisotropy *a* d *ij* that describes the anisotropy arising from the distribution of branch vectors (vectors connecting the centers of contacting particles):

$$\begin{aligned} a_{ij}^{d}=\frac{15}{2}\frac{d_{ij}^{'}}{d^{0}}\#\left( S7 \right) \end{aligned}$$

where *d ' ij* is the deviatoric part of *d_ij_*, *d*^0^ is a normalization factor calculated from the trace of *d_ij_* defined by:

$$\begin{aligned} d_{ij}=\frac{1}{N_{c}}\sum_{c\in N_{c}} \frac{d^{c}n_{i}n_{j}}{1+a_{kl}^{c}n_{k}n_{l}}\#\left( S8 \right) \end{aligned}$$

where *d*^c^ is the magnitude of the branch vector for a single contact *c*, and *a* c *kl* is the pre-calculated contact fabric anisotropy tensor.

The third one is normal force anisotropy *a* n *ij* that quantifies the anisotropy in the distribution of normal contact forces:

$$\begin{aligned} a_{ij}^{n}=\frac{15}{2}\frac{Z_{ij}^{n^{'}}}{\bar{f^{0}}}\#\left( S9 \right) \end{aligned}$$

where *Z*n' *ij* is the deviatoric part of *Z* n *ij*, $\bar{f^{0}}$ is a normalization factor calculated from the trace of *Z* n *ij* calculated as:

$$\begin{aligned} Z_{ij}^{n}=\frac{1}{N_{c}}\sum_{c\in N_{c}} \frac{f^{n}n_{i}n_{j}}{1+a_{kl}^{c}n_{k}n_{l}}\#\left( S10 \right) \end{aligned}$$

where *f* ^n^ is the magnitude of the normal contact force at a single contact *c*.

The fourth one is tangential force anisotropy *a* t *ij* that describes the anisotropy arising from the distribution of tangential (frictional) contact forces:

$$\begin{aligned} a_{ij}^{t}=\frac{15}{3}\frac{Z_{ij}^{t^{'}}}{\bar{f^{0}}}\#\left( S11 \right) \end{aligned}$$

where *Z* t' *ij* is the deviatoric part of *Z* t *ij* calculated as:

$$\begin{aligned} Z_{ij}^{t}=\frac{1}{N_{c}}\sum_{c\in N_{c}} \frac{f^{t}t_{i}n_{j}}{1+a_{kl}^{c}n_{k}n_{l}}\#\left( S12 \right) \end{aligned}$$

where *f* ^t^ is the magnitude of the tangential contact force at a single contact *c*, *t_i_* is the unit tangential vector at the contact plane.

A scalar representation of anisotropy provides a more intuitive understanding. The signed magnitude of anisotropy *a*^*^ represents both the intensity of the anisotropy and its orientation relative to the applied stress:

$$\begin{aligned} a^{*}=\text{sign}\left( S_{r} \right)\sqrt{\frac{3}{2}a_{ij}^{*}a_{ij}^{*}}\#\left( S13 \right) \end{aligned}$$

where ^*^ can be c, d, n, or t, and *a* * *ij* refers to one of the four anisotropy types.

Coaxiality factor *S*_r_ measures the degree of alignment between an anisotropy tensor and the deviatoric stress tensor:

$$\begin{aligned} S_{r}=\frac{a_{ij}^{*}\sigma_{ij}^{'}}{\sqrt{a_{kl}^{*}a_{kl}^{*}}\sqrt{\sigma_{mn}^{'}\sigma_{mn}^{'}}}\#\left( S14 \right) \end{aligned}$$

where *σ* ' *ij* is the deviatoric part of the applied stress tensor *σ_ij_*, and *σ_ij_* is calculated by averaging the microscopic forces over a representative volume:

$$\begin{aligned} \sigma_{ij}=\frac{1}{V}\sum_{c\in N_{c}} f_{i}^{c}d_{j}^{c}\#\left( S15 \right) \end{aligned}$$

where *V* is the representative volume, *N*_c_ is the total number of contacts within the volume, *f* c *i* is the *i*-th component of the total contact force vector at contact *c*, and *d* c *j*is the *j*-th component of the branch vector at contact *c*.


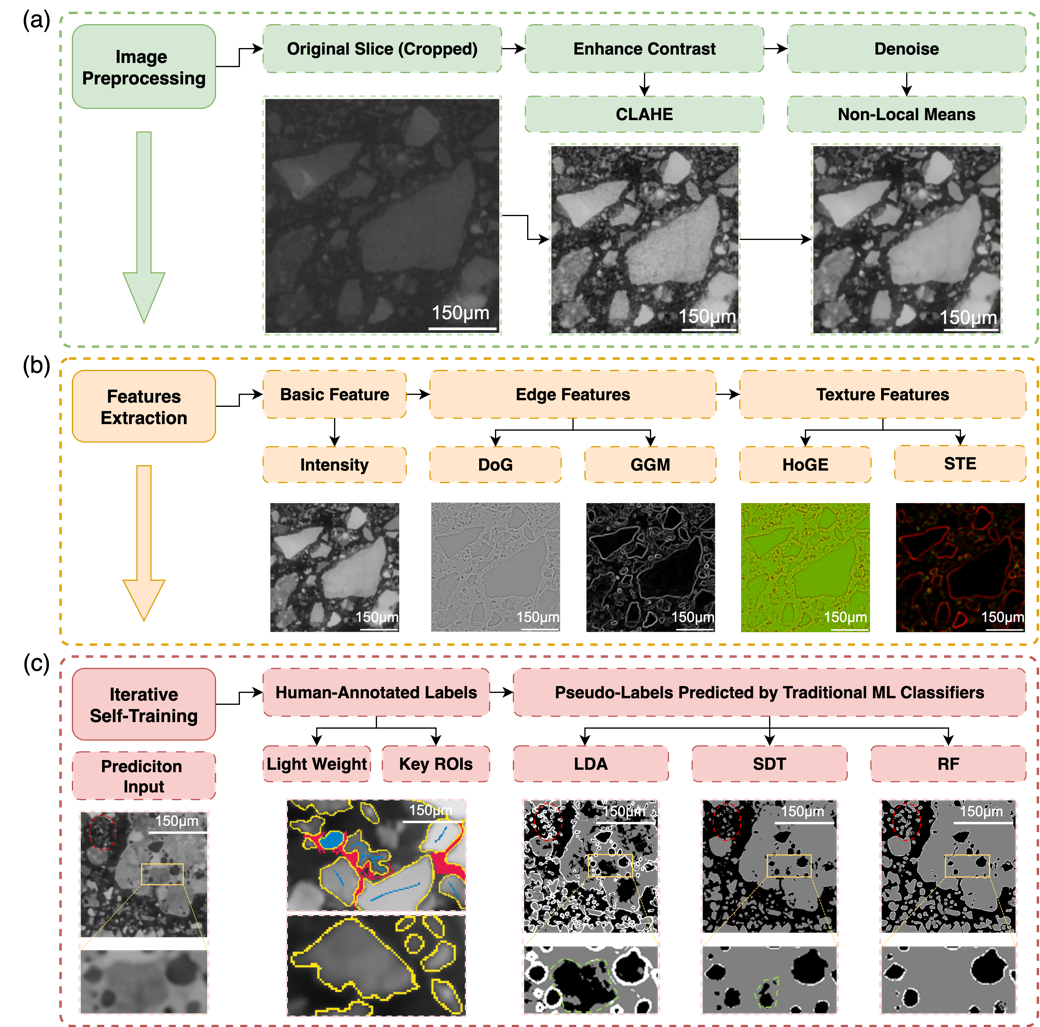


**Figure S1** Schematic of the iterative self-training framework for semantic segmentation of lunar regolith particles from micro-CT images. (a) Image Preprocessing: The raw micro-CT slices undergo contrast enhancement using CLAHE algorithm and are subsequently denoised with NLM filter. (b) Feature Extraction: A multi-dimensional feature set is engineered, comprising basic intensity, edge features (DoG, GGM), and texture features (HoGE, STE). (c) Iterative Self-Training: A semi-supervised learning process is initiated using a small set of human-annotated labels. These labels are used to train traditional machine learning classifiers, which in turn generate pseudo-labels for the unlabeled data. The model is then iteratively refined by retraining on these pseudo-labels.


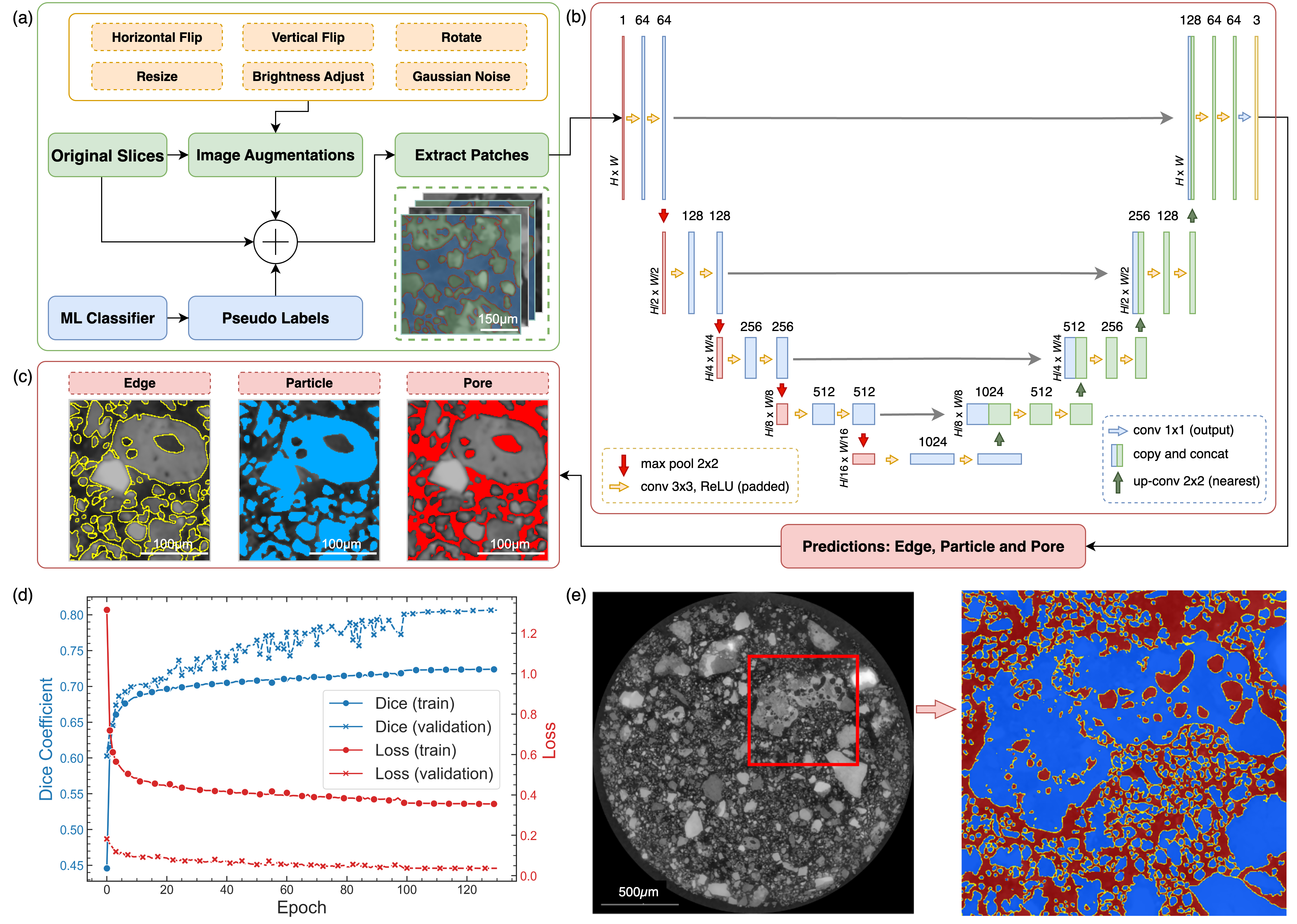


**Figure S2** U-Net framework and performance for multi-class semantic segmentation of lunar regolith. (a) Data preparation and augmentation pipeline. The raw micro-CT slices are combined with pseudo-labels, augmented (e.g., flips, rotation, brightness adjustment), and then partitioned into patches for model training. (b) Schematic of the U-Net architecture employed in this study. (c) Example multi-class predictions from the trained model, simultaneously identifying particle edges (yellow), bodies (blue), and pore space (red). (d) Training and validation curves, plotting the dice coefficient (left axis) and loss (right axis) as a function of training epoch. (e) Qualitative segmentation result on a representative region of a single 2D micro-CT slice, demonstrating the model's effectiveness in delineating particles and pores in a morphologically complex region.


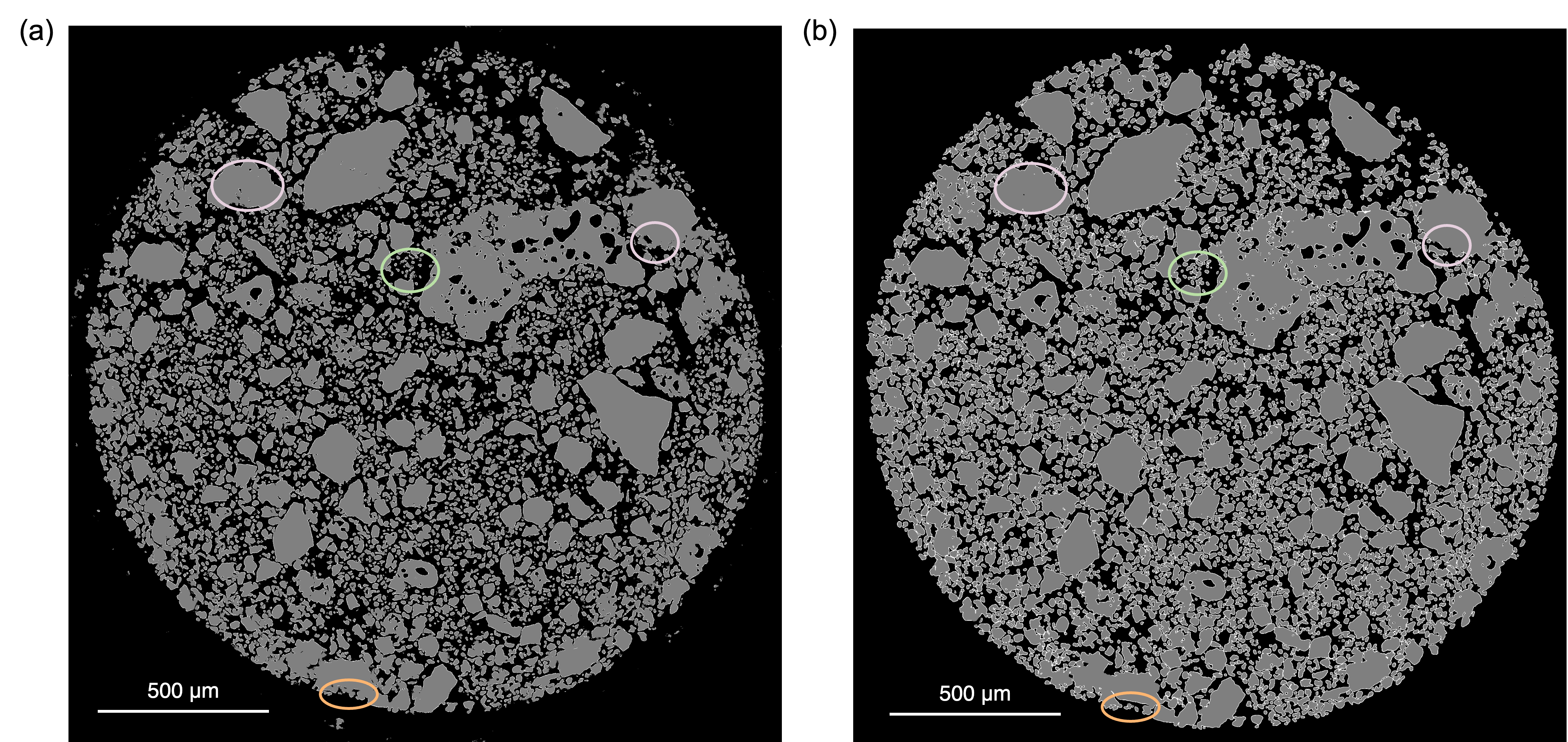


**Figure S3** Comparison of segmentation performance between RF and U-Net approaches. Cross-sectional segmentation results showing (a) RF pseudo-labels and (b) refined U-Net predictions, where white, gray, and black pixels represent boundaries, particles, and pore spaces, respectively. The colored circles highlight key improvements achieved by U-Net: enhanced recognition of small particles (green), improved boundary continuity and accuracy for complex particle contours (orange), and elimination of false void predictions within particle interiors (purple).


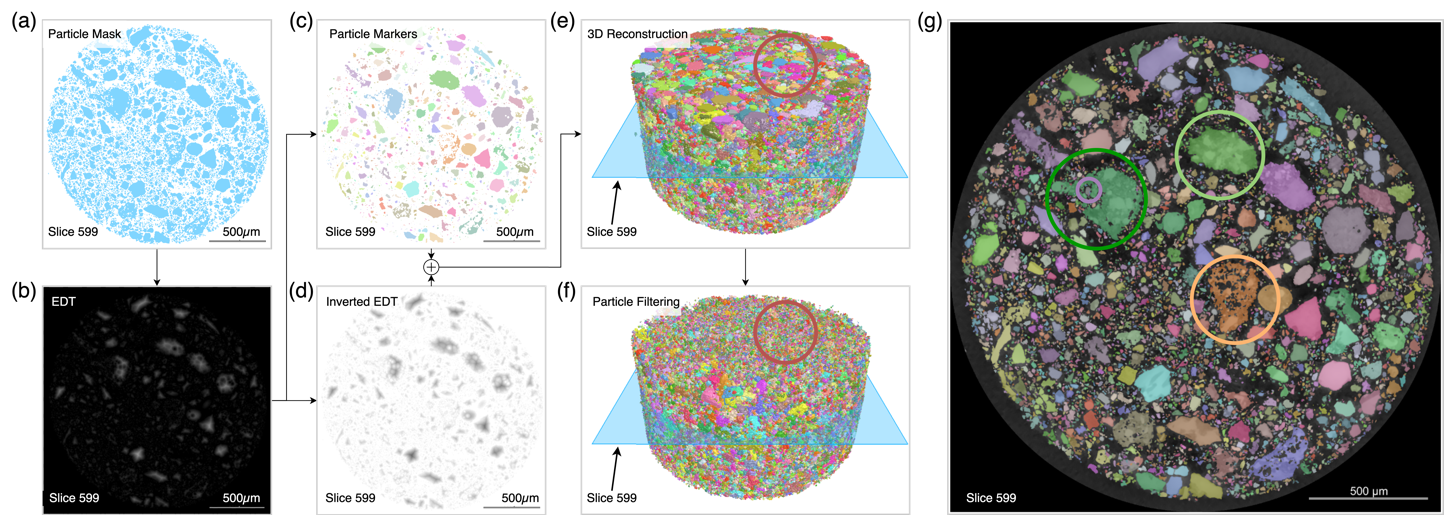


**Figure S4** Instance segmentation of individual particles on a representative 2D slice using a marker-controlled watershed algorithm. (a-d) Steps of the watershed algorithm applied to every single slice. (a) Binary particle mask generated by the U-Net model, which serves as the input for the segmentation. (b) EDT computed from the binary mask, where intensity is proportional to the distance from the nearest boundary. (c) Local maxima identified from the EDT, serving as markers for the cores of individual particles. (d) The inverted EDT, which creates the topographic landscape for the watershed transform. (e, f) 3D rendering of the particle collective, where the blue plane indicates the position of the 2D slice shown in panels (a-d, g). (e) Initial 3D rendering of the segmented particle instances prior to post-processing. (f) Final 3D rendering of the particle collective after filtering to remove fragments and particles intersecting the volume boundary. (g) The final 2D instance segmentation for the selected slice showing the final instance segmentation (colored particles) overlaid on the raw micro-CT image, demonstrating successful separation of particles in intensity heterogeneity particles (green circles) and accurate delineation of grains with internal porosity (orange circles).

**Figure S5** Quality control in 2D particle size analysis from BSE imaging. Comparison of (a) raw BSE image patch and (b) filtered particle dataset where only complete particles (colored) are retained for size analysis, with edge-touching particles (grayscale) systematically excluded to prevent bias from incomplete particle boundaries.


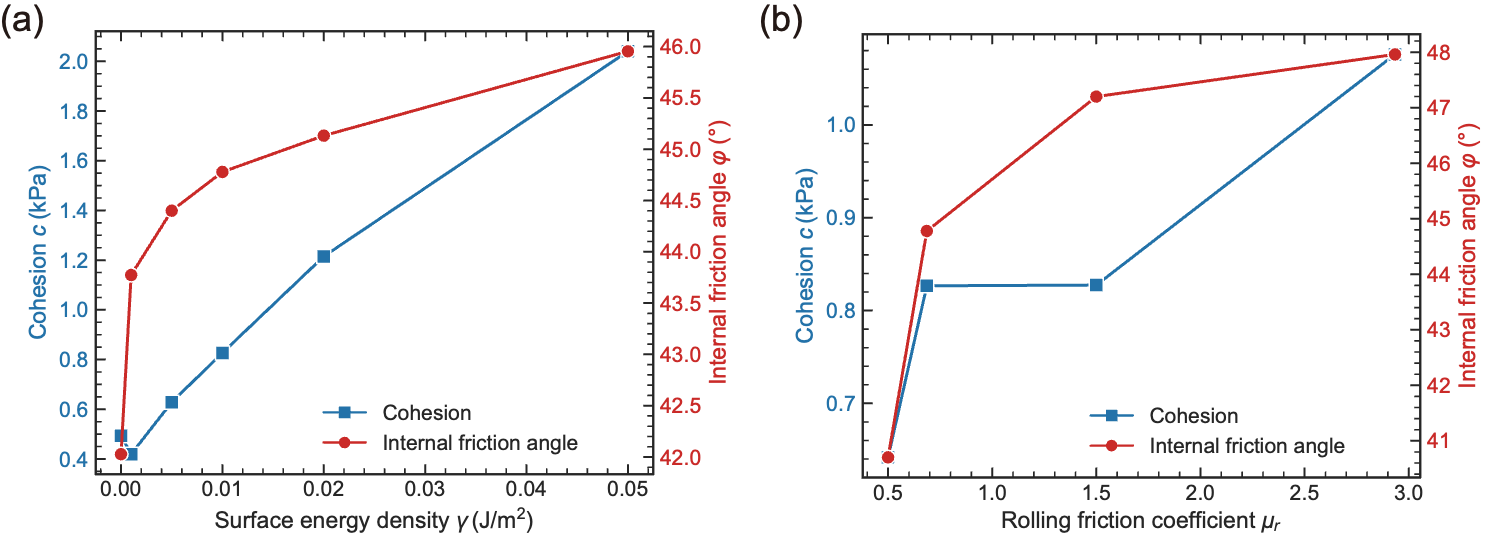


**Figure S6** Parameter sensitivity analysis of DEM triaxial compression tests showing the decoupled effects of particle morphology and adhesion. (a) Effect of surface energy (*γ*) on shear strength (at fixed *μ*_r_ = 0.7), showing *γ* is the dominant factor controlling cohesion (*c*). (b) Effect of rolling resistance (*μ*_r_) on shear strength (at fixed *γ* = 0.01 J/m²), showing *μ*_r_ is the dominant factor controlling the internal friction angle (*φ*). This analysis quantitatively isolates the net contribution of the CE-6 morphology. A baseline model (*μ*_r_ = 0.7) at common adhesion (*γ* = 0.01 J/m²) yields *φ*_baseline_ ≈ 44.78°. In contrast, the CE-6 model (using the measured shape proxy, *μ*_r_ ≈ 2.9) at the same adhesion level yields *φ*_paper_ ≈ 47.96°. This demonstrates that the net contribution (Δ*φ*) from the CE-6 particle morphology is 3.18°. This morphology-driven gain in *φ* contributes to the total shear strength *τ* = *σ*·tan*φ* + *c* as a multiplicative term (*σ*·tan*φ*), confirming it as the dominant factor responsible for the high shear strength observed in our simulations.


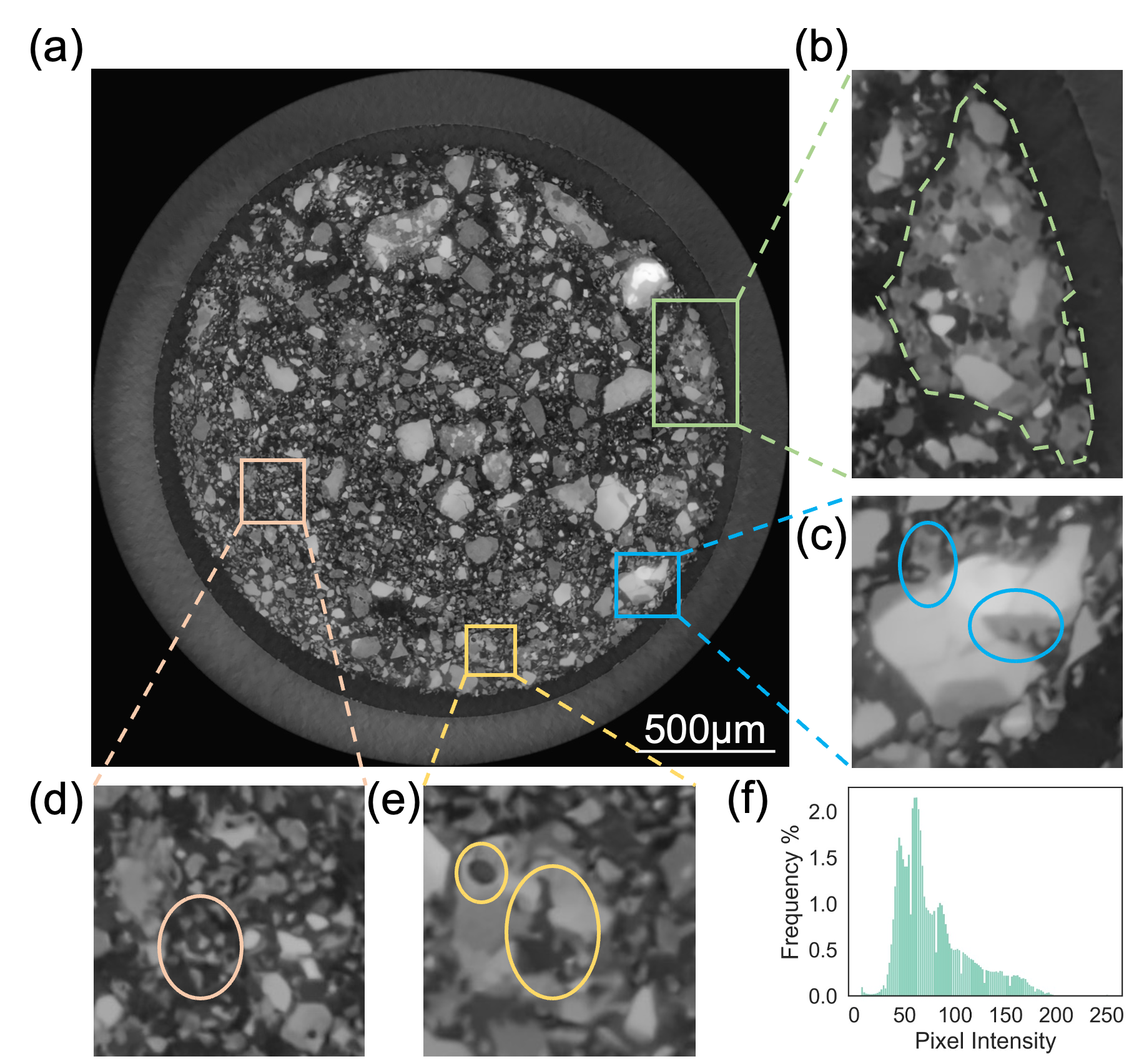


**Figure S7** Challenges in automated segmentation of lunar regolith micro-CT images. (a) Overview of the CE-6 lunar regolith sample showing heterogeneous particle distribution. Representative challenges include: (b) complex particle morphology with irregular boundaries, (c) multi-mineral particles with varying grayscale contrasts, (d) presence of fine particles, (e) intra-particle porosity within individual grains, and (f) continuous grayscale histogram without distinct peaks, complicating threshold-based segmentation approaches.


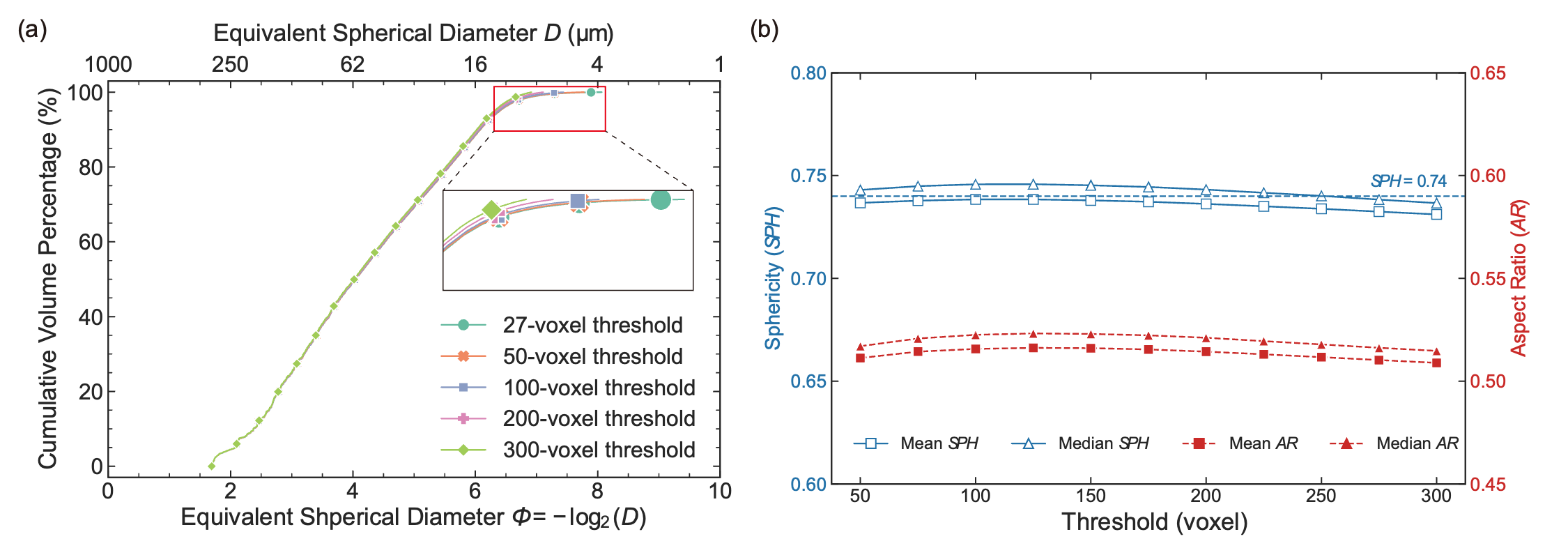


**Figure S8** Sensitivity analysis of morphological statistics to variations in the particle volume filtering threshold. (a) GSD curves calculated using 5 different volume thresholds (ranging from 27 to 300 voxels). The curves exhibit high consistency, with only minor deviations in the finest fraction, indicating the stability of the distribution. (b) Variation of the mean and median *SPH* and *AR* across the same range of thresholds. The statistical metrics remain highly stable (e.g., mean *SPH* stays at about 0.74) as the threshold increases, demonstrating that the observed high irregularity of CE-6 particles is a robust feature and not an artifact of small-particle voxelization errors.


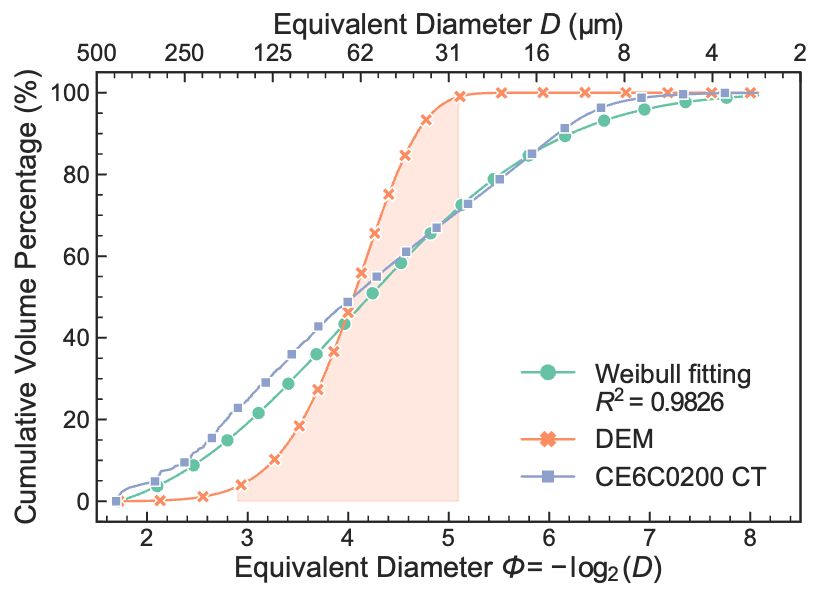


**Figure S9** Generation of the representative grain size distribution for DEM simulation. The plot compares the GSD from the micro-CT measurements (CE6C0200 CT) with the distribution used in the DEM simulations. The DEM GSD was derived by first fitting a two-parameter Weibull distribution to the experimental data (*R*² = 0.9826). Subsequently, the numerical GSD was generated using this function, constrained by adopting the experimental *d*_50_ and selecting a shape parameter of 7. The shaded region indicates the range of particle sizes encompassed by the DEM model, which effectively captures the key percentiles (*d*_30_, *d*_50_, and *d*_60_) of the actual regolith while ensuring computational feasibility.

**Table S1** Statistical Summary of CE-6 Morphological Parameters

| Parameter | Min | Max | Mean | Median | Mode |
| --- | --- | --- | --- | --- | --- |
| *ESD* (µm) | 3.74 | 309.65 | 11.62 | 10.03 | 3.83 |
| *MFD* (µm) | 4.18 | 457.74 | 15.98 | 13.03 | 5.8 |
| *SPH* | 0.16 | 0.99 | 0.74 | 0.74 | 0.95 |
| *AR* | 0.01 | 0.96 | 0.50 | 0.51 | 0.48 |
| *TRP* (µm) | 0.00 | 257.70 | 7.47 | 5.69 | - |

**Table S2** Mean Shape Parameters per Particle Size Class

| Size Class (Wentworth, 1922)^[37]^ | Mean *SPH* | Mean *AR* |
| --- | --- | --- |
| Coarse Clay | 0.74 | 0.378 |
| Very Fine Silt | 0.74 | 0.496 |
| Fine Silt | 0.76 | 0.514 |
| Medium Silt | 0.67 | 0.500 |
| Coarse Silt | 0.60 | 0.529 |
| Very Fine Sand | 0.51 | 0.521 |
| Fine Sand | 0.43 | 0.509 |
| Medium Sand | 0.43 | 0.463 |

**Table S3** List of Abbreviations

| Abbreviation | Full Name | Brief Explanation |
| --- | --- | --- |
| 2D / 3D | Two-Dimensional / Three-Dimensional | Refers to the number of spatial dimensions. |
| *AR* | Aspect Ratio | A shape parameter describing the ratio of a particle's shortest to longest principal axis. |
| BSE | Backscattered Electron | An imaging mode in Scanning Electron Microscopy (SEM) sensitive to elemental composition. |
| CE-5 / CE-6 | Chang'e-5 / Chang'e-6 | Chinese Lunar Exploration Program missions. |
| CLAHE | Contrast-Limited Adaptive Histogram Equalization | An image processing algorithm to enhance local contrast. |
| CT | Computed Tomography | A non-destructive 3D imaging technique using X-rays. |
| *C*_u_ / *C*_c_ | Coefficient of Uniformity / Coefficient of Curvature | Geotechnical parameters describing the shape of a grain size distribution curve. |
| *d*_n_ | Characteristic Particle Diameter | The particle diameter at which n% of the sample's volume is composed of finer particles. |
| DEM | Discrete Element Method | A numerical method for computing the motion and effect of a large number of particles. |
| DoG | Difference of Gaussian | An image processing algorithm for edge detection. |
| DT | Decision Tree | A supervised machine learning algorithm. |
| EDT | Euclidean Distance Transform | An image processing operation that calculates the distance from every pixel to the nearest non-zero pixel. |
| *ESD* (*D*_s_) | Equivalent Spherical Diameter | The diameter of a sphere that has the same volume as the particle. |
| GGM | Gaussian Gradient Magnitude | An image processing algorithm for edge detection. |
| GSD | Grain Size Distribution | The distribution of particle sizes presents in a granular material. |
| HoGE | Histogram of Oriented Gradient | A feature descriptor used in computer vision for object detection. |
| ISRU | In-Situ Resource Utilization | The practice of collecting, processing, and using materials found on other astronomical objects. |
| JKR | Johnson-Kendall-Roberts | A contact mechanics model that accounts for adhesive forces between particles. |
| LDA | Linear Discriminant Analysis | A supervised machine learning algorithm for classification. |
| *MFD* (*D*_F_) | Mean Feret Diameter | A measure of particle size based on the average of caliper measurements over many orientations. |
| NLM | Non-Local Means | An image processing algorithm for noise reduction. |
| RF | Random Forest | An ensemble supervised machine learning algorithm. |
| *SA* | Surface Area | The total area of the particle's surface. |
| SEM | Scanning Electron Microscope | A type of electron microscope that produces images of a sample by scanning the surface with a focused beam of electrons. |
| SPA | South Pole-Aitken (Basin) | A large impact basin on the far side of the Moon, where the CE-6 sample was collected. |
| *SPH* | 3D Sphericity | A shape parameter quantifying how closely a particle's shape resembles a perfect sphere. |
| STE | Statistical Texture Extraction | A set of features used to quantify the texture of an image region. |
| *TRP* | Total Roughness Proxy | A parameter used in this study to quantify the overall, large-scale surface undulation of a particle. |
| *VOL* | Volume | The total volume occupied by a particle. |

Note: Abbreviations for physical parameters are italicized; those for methods, models, and missions are shown in standard font.

**Table S4** Seven 3D morphological characteristics of CE-6 Lunar Regolith (Please refer to the attachments).

1. High-resolution visual validation of the multi-stage segmentation pipeline on a representative micro-CT slice.

Due to its high resolution and large file size, this figure is provided as a separate supplementary data file and is not embedded within the main manuscript. This figure provides a detailed, high-resolution comparison of the segmentation results on a single, representative micro-CT slice (No. 599) to illustrate the pipeline's performance.

**(A)** The raw, high-resolution micro-CT slice (2516×2516 pixels), with the central 2084×2084 pixels region used for analysis indicated by the red box. Magnified insets show four common segmentation challenges present in the regolith sample: **(A.1)** a particle with complex edges, **(A.2)** a multi-mineral grain exhibiting varying grayscale contrast, **(A.3)** a dense cluster of fine and contacting particles, and **(A.4)** a large particle containing significant internal porosity. The colored frames of the insets correspond to the locations shown in the main image.

**(B)** The corresponding pixel-wise semantic segmentation result, shown as a semi-transparent overlay on the raw micro-CT image. Pixels are classified as pore (red), particle (blue), or edge (yellow). The magnified views demonstrate the high accuracy of the U-Net model in precisely delineating complex boundaries (often only 2-3 pixels wide) and successfully identifying the challenging features highlighted in panel A. This validates the effectiveness of the multi-class segmentation approach, especially for small and contacting particles.

**(C)** The final particle instance segmentation result after applying a marker-controlled watershed algorithm, where each distinct color represents an individual particle instance. The method successfully separates most particles, including the challenging, densely packed fine grains (**C.3**). However, it also illustrates a known limitation of the standard marker-based watershed algorithm: the internal porosity of complex brecciated particles can generate multiple seed points, leading to over-segmentation where a single particle is erroneously subdivided (**C.4**). This is an inherent issue with this algorithm and a common challenge in the field.

In summary, this figure demonstrates that the primary methodological contribution of our pipeline is the highly accurate semantic segmentation, which provides a robust foundation for subsequent analysis. While the standard watershed algorithm has limitations for certain particle types—which could be addressed in future work with more advanced, boundary-based methods—our multi-layered validation (expert guidance, iterative training, and independent BSE cross-verification) confirms that the overall process yields a high-fidelity reconstruction suitable for the large-scale statistical analysis presented in this study.
