# Supplementary figures and images for: Particle Morphology Controls the Bulk Mechanical Behavior of Far-Side Lunar Regolith from Chang’e-6 Samples and Deep Learning
Source: Research (Wash D C). 2026 Jan 8;9:1064. doi: 10.34133/research.1064 (PMC12779894; doi:10.34133/research.1064)

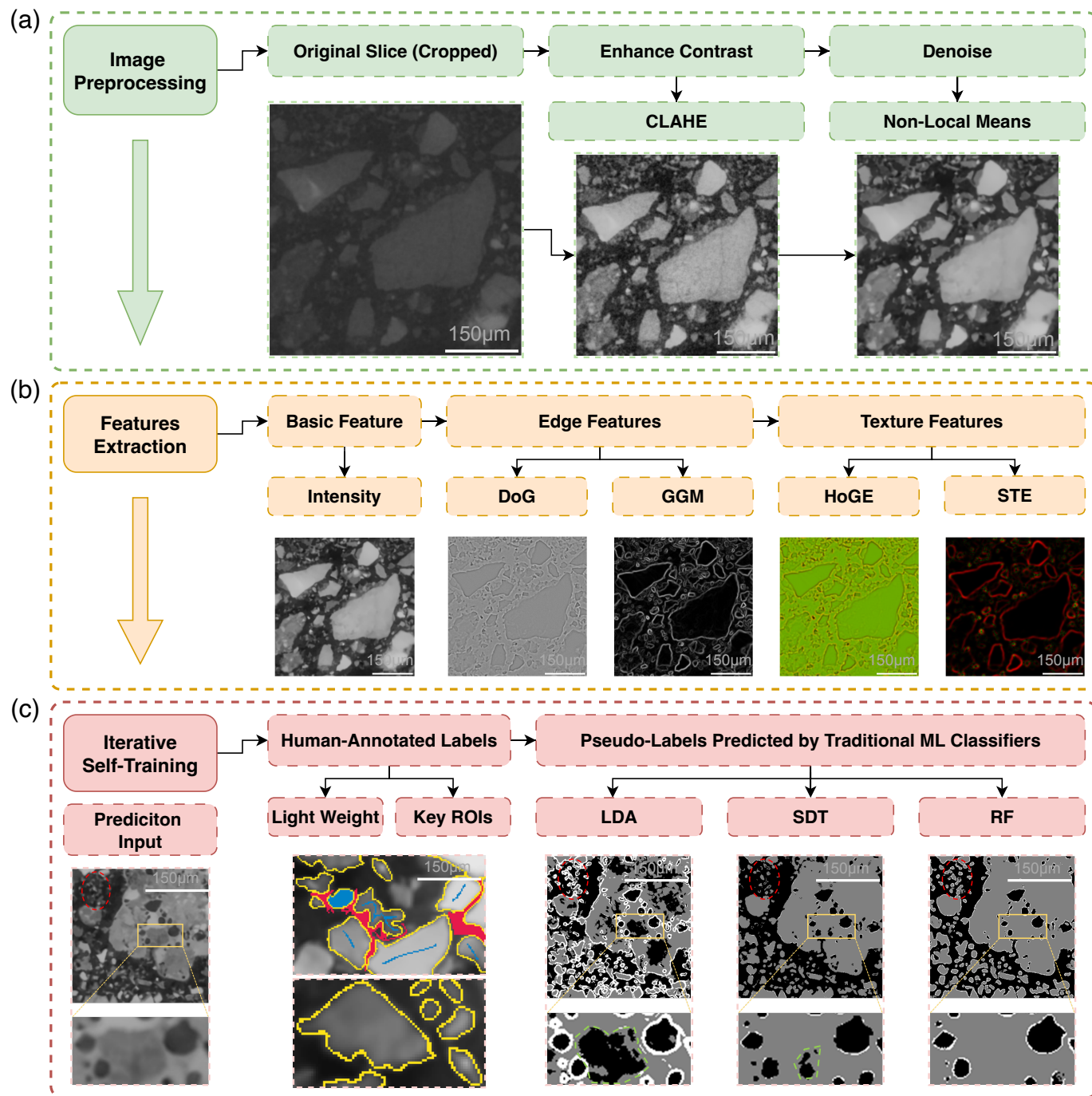

Supplement: Supplementary 1 — Notes S1 to S6 Figs. S1 to S9 Tables S1 to S4 Data File S1 [file research.1064.f1.zip › Figure S1.pdf]

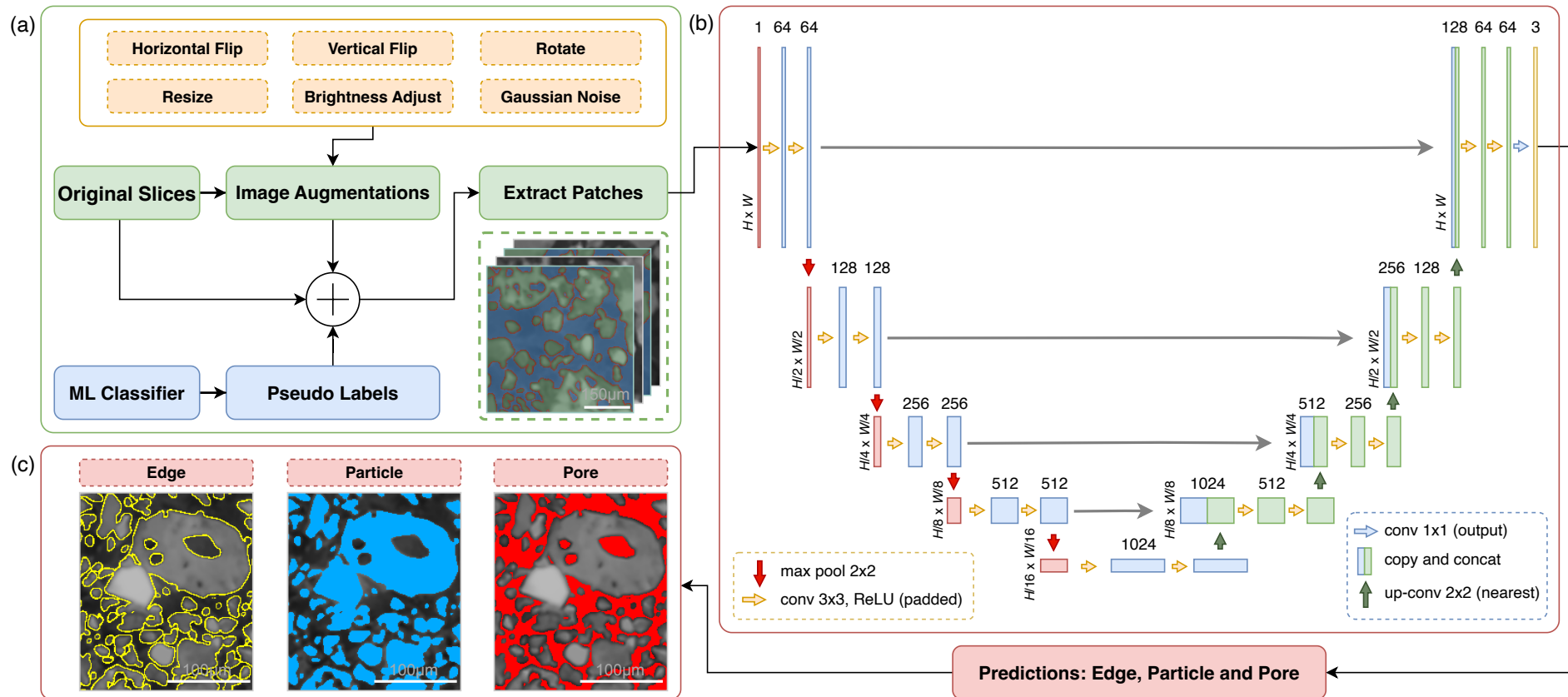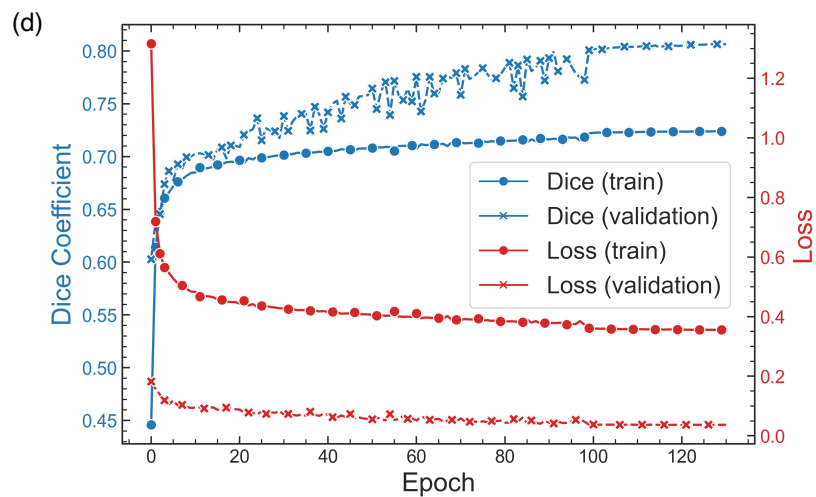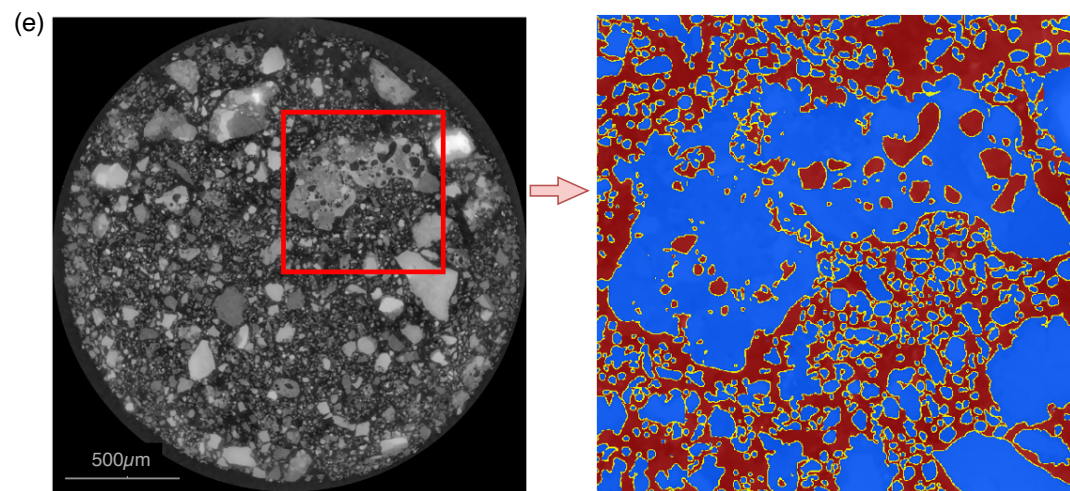

Supplement: Supplementary 1 — Notes S1 to S6 Figs. S1 to S9 Tables S1 to S4 Data File S1 [file research.1064.f1.zip › Figure S2.pdf]

(a)

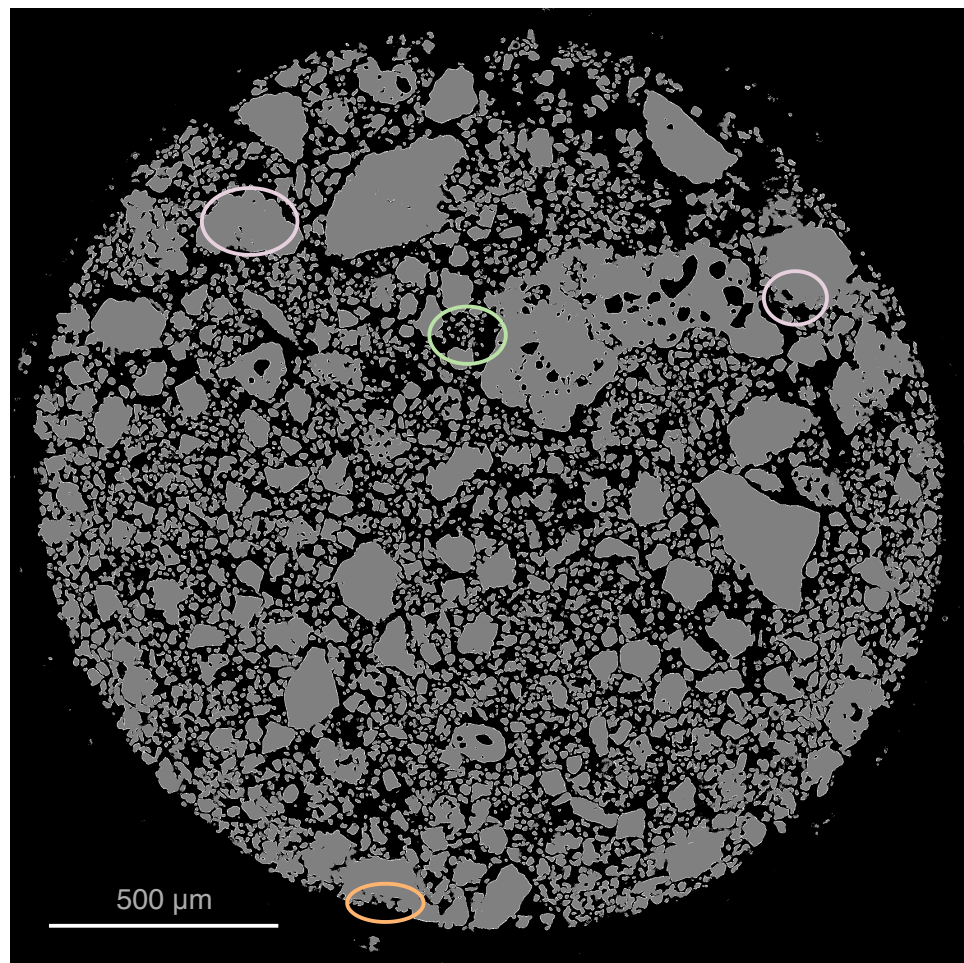

(b)

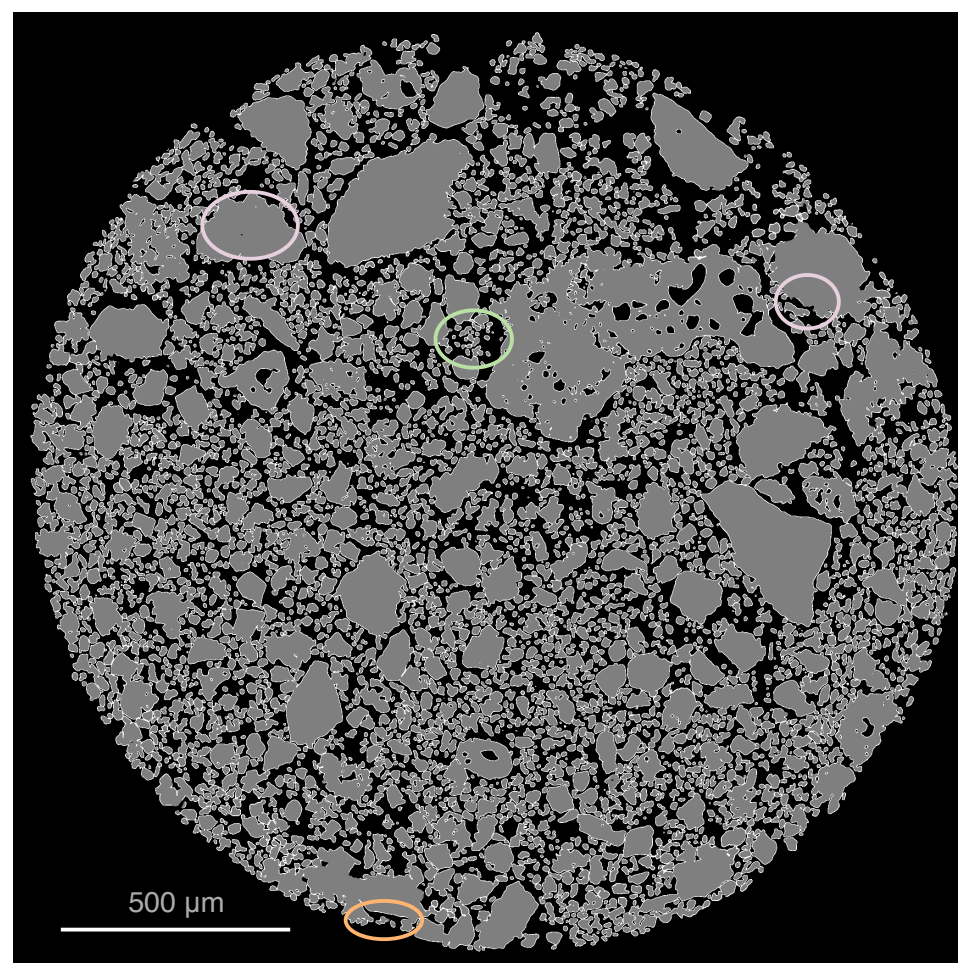

Supplement: Supplementary 1 — Notes S1 to S6 Figs. S1 to S9 Tables S1 to S4 Data File S1 [file research.1064.f1.zip › Figure S3.pdf]

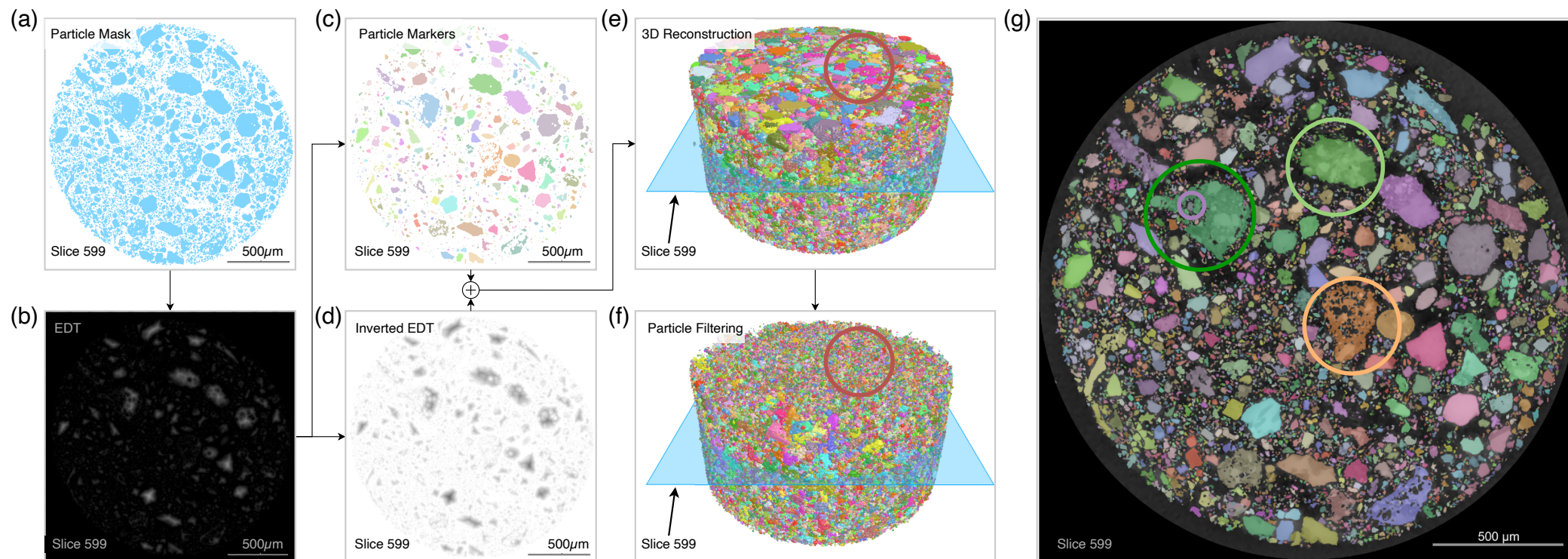

Supplement: Supplementary 1 — Notes S1 to S6 Figs. S1 to S9 Tables S1 to S4 Data File S1 [file research.1064.f1.zip › Figure S4.pdf]

(a)

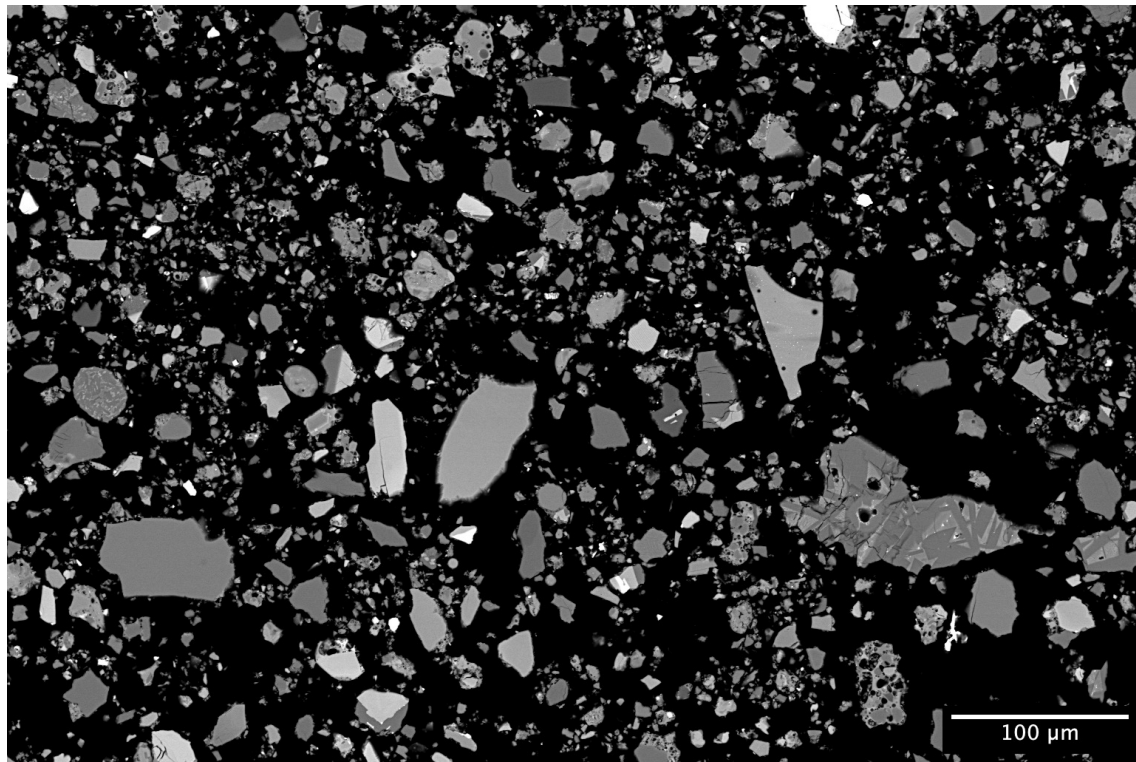

(b)

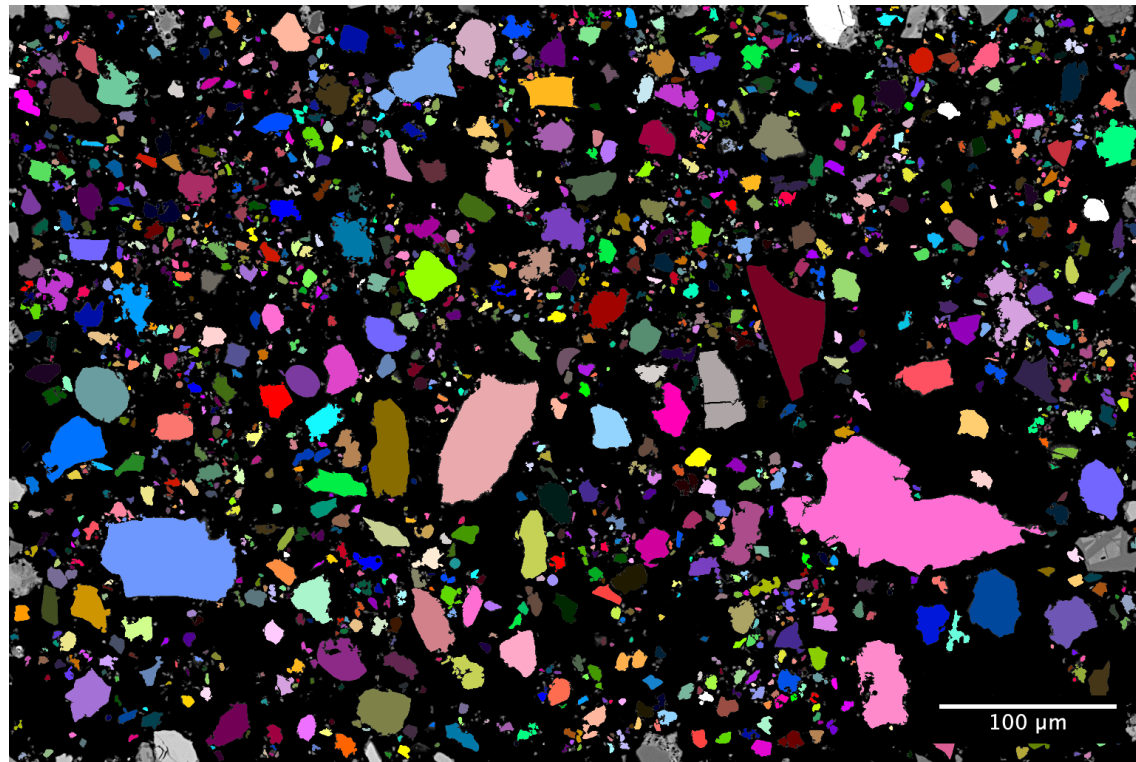

Supplement: Supplementary 1 — Notes S1 to S6 Figs. S1 to S9 Tables S1 to S4 Data File S1 [file research.1064.f1.zip › Figure S5.pdf]

(a)

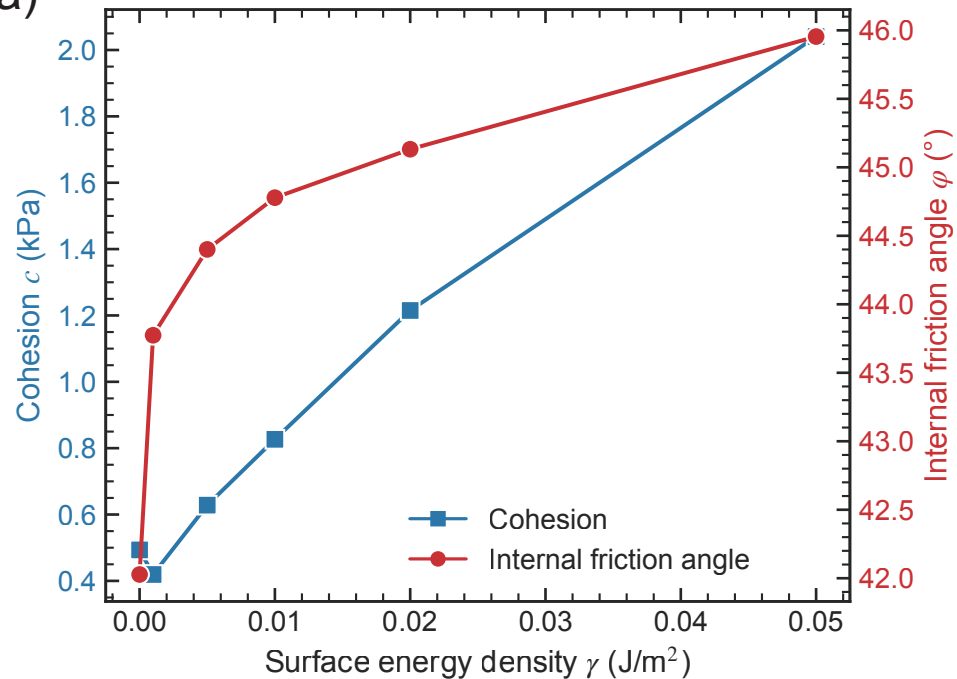

(b)

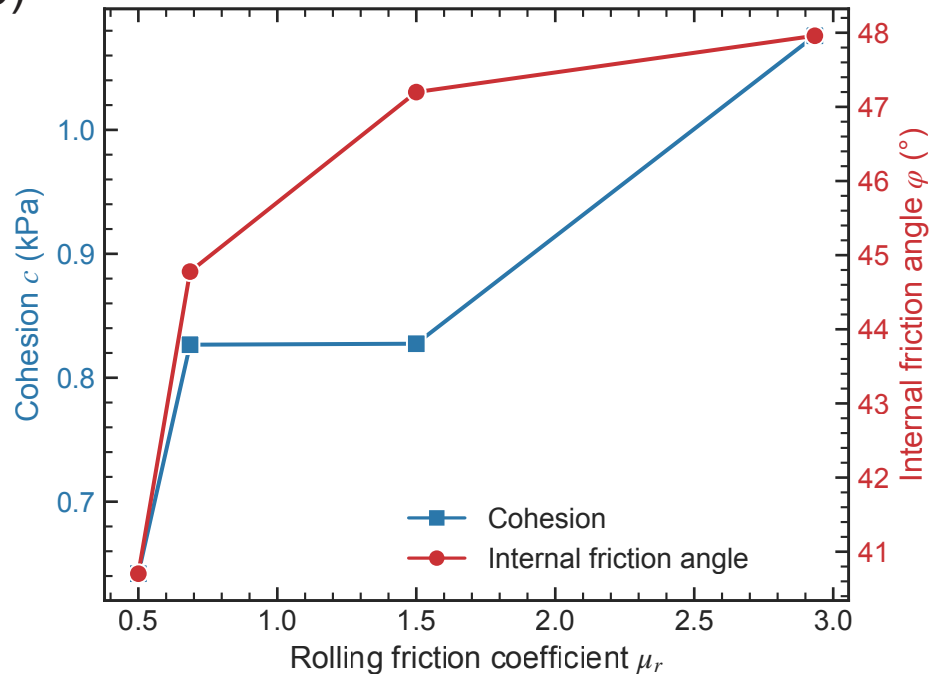

Supplement: Supplementary 1 — Notes S1 to S6 Figs. S1 to S9 Tables S1 to S4 Data File S1 [file research.1064.f1.zip › Figure S6.pdf]

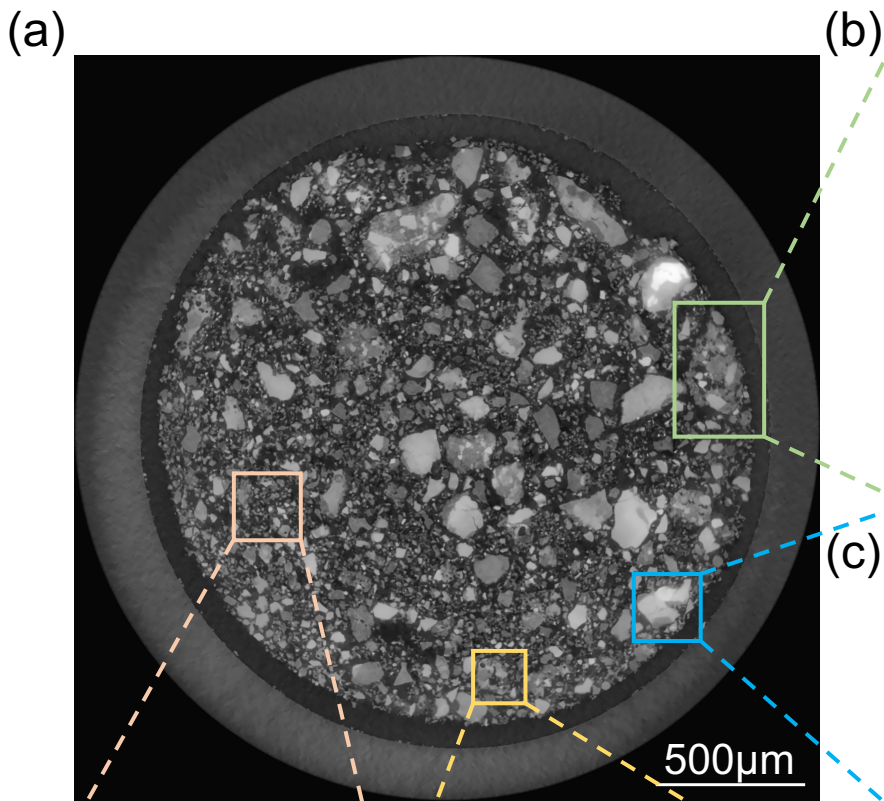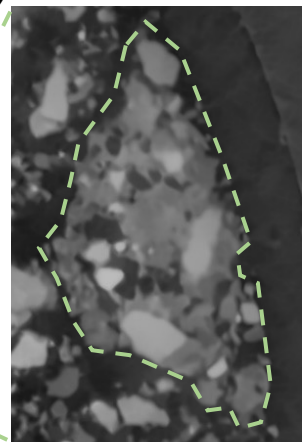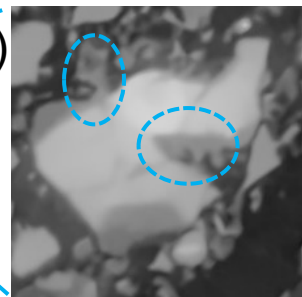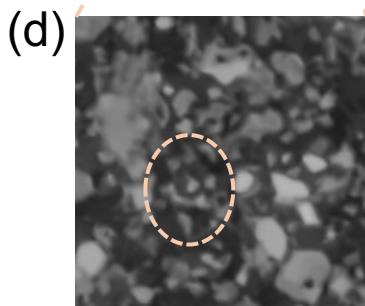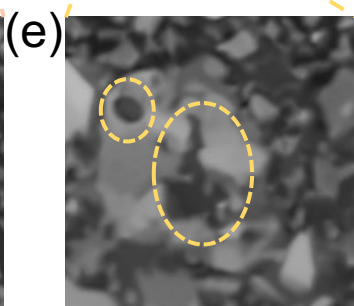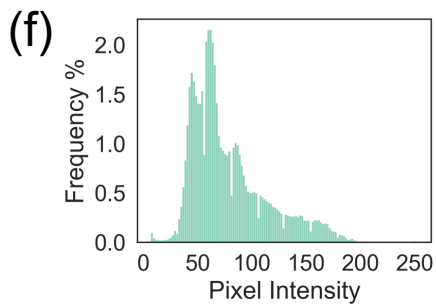

Supplement: Supplementary 1 — Notes S1 to S6 Figs. S1 to S9 Tables S1 to S4 Data File S1 [file research.1064.f1.zip › Figure S7.pdf]

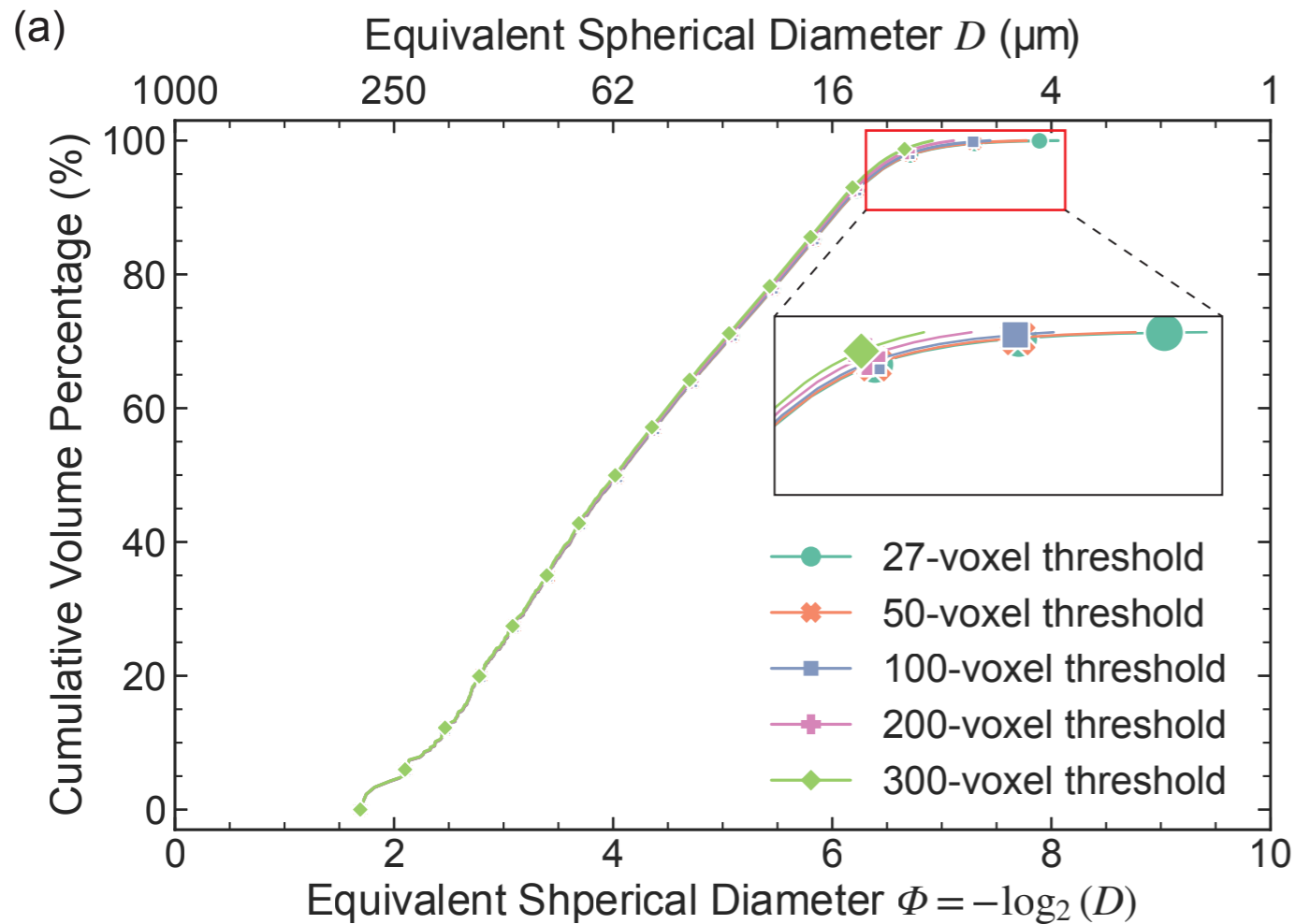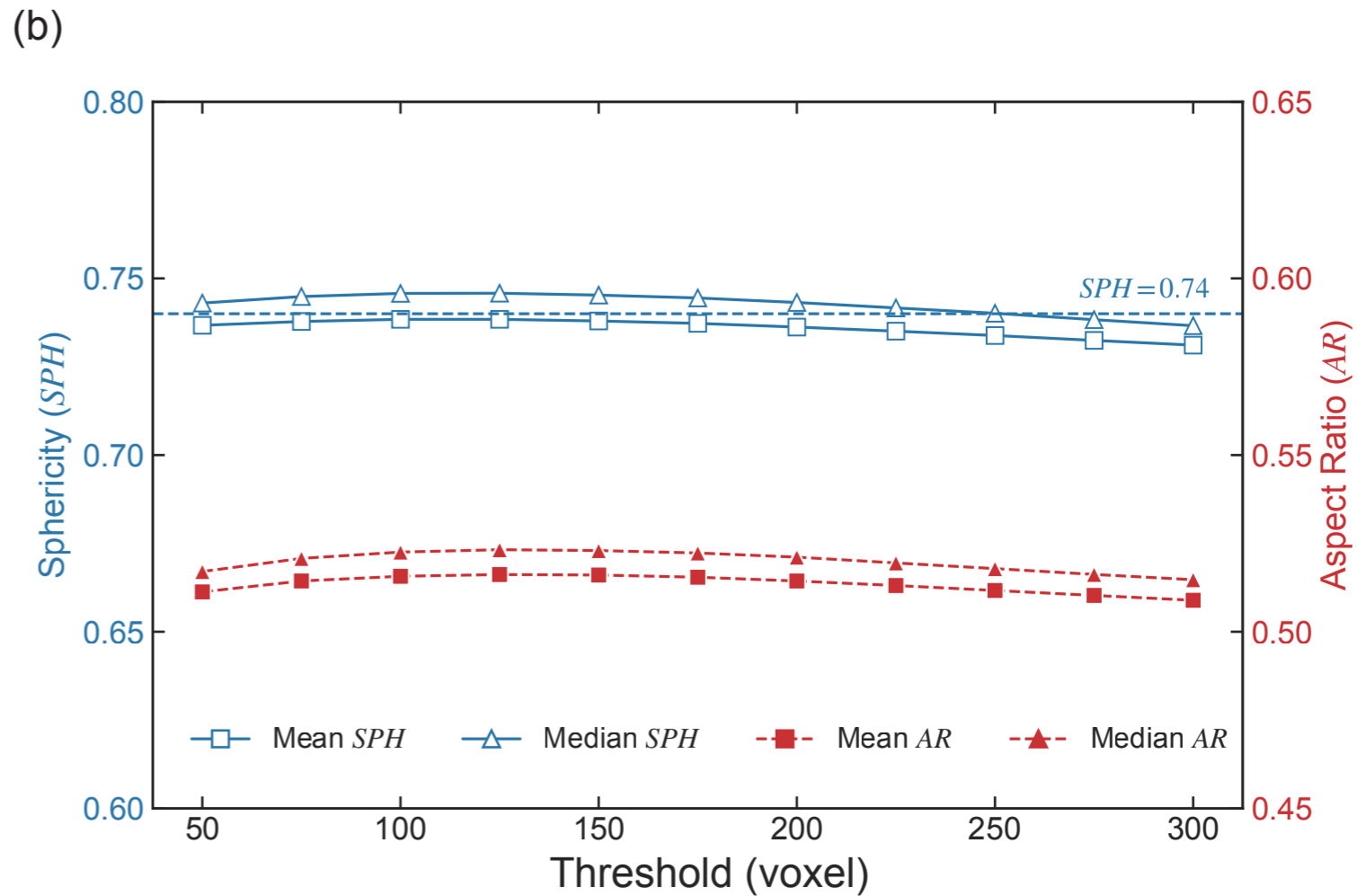

Supplement: Supplementary 1 — Notes S1 to S6 Figs. S1 to S9 Tables S1 to S4 Data File S1 [file research.1064.f1.zip › Figure S8.pdf]

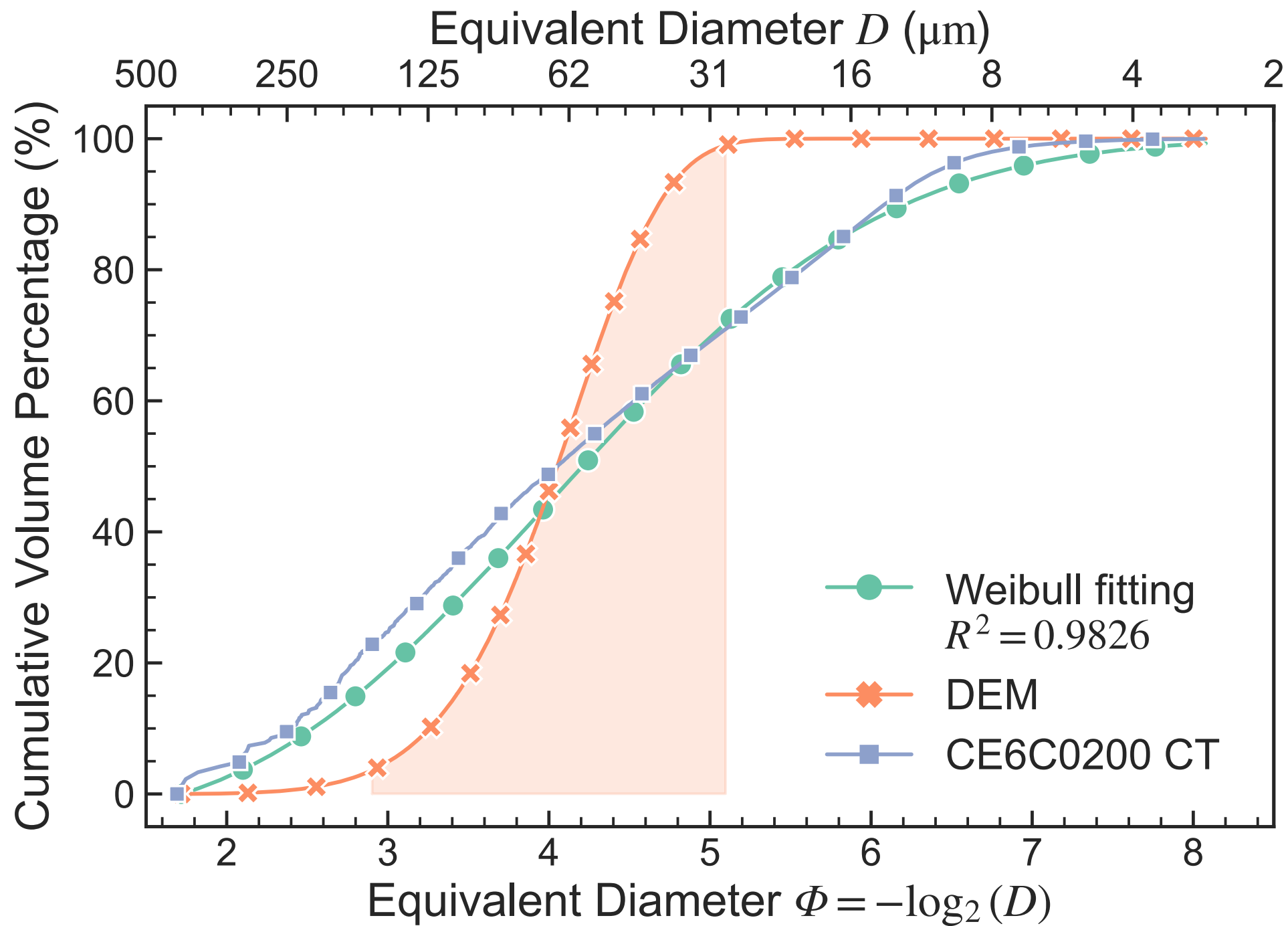

Supplement: Supplementary 1 — Notes S1 to S6 Figs. S1 to S9 Tables S1 to S4 Data File S1 [file research.1064.f1.zip › Figure S9.pdf]
