# Supplementary material for: Particle Morphology Controls the Bulk Mechanical Behavior of Far-Side Lunar Regolith from Chang’e-6 Samples and Deep Learning
Source: Research (Wash D C). 2026 Jan 8;9:1064. doi: 10.34133/research.1064 (PMC12779894; doi:10.34133/research.1064)
Supplement: Supplementary 1 — Notes S1 to S6 Figs. S1 to S9 Tables S1 to S4 Data File S1 [file Data file S1.pdf › Data file S1.pdf]

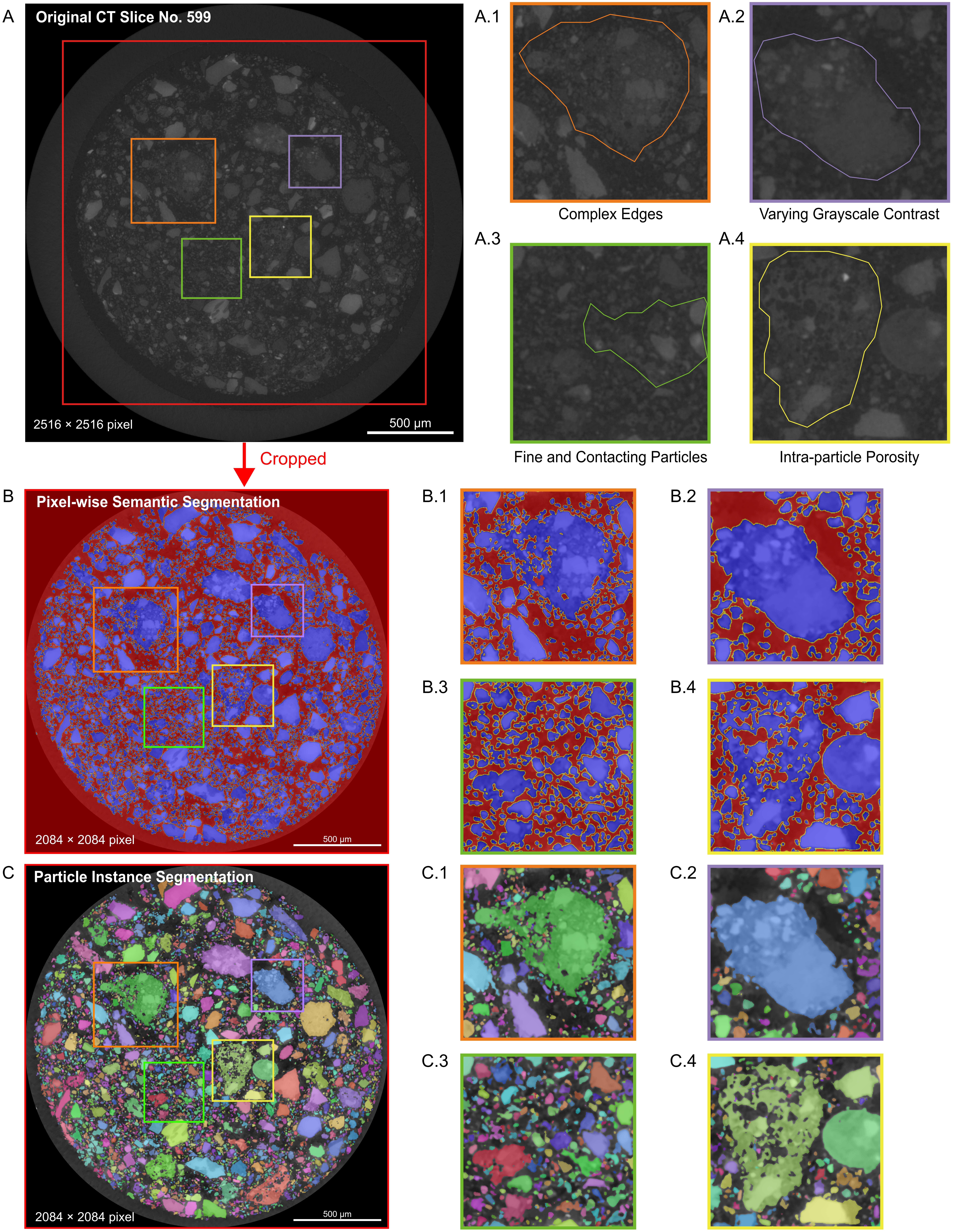

A
Original CT Slice No. 599
2516 × 2516 pixel
500 µm
A.1
Complex Edges
A.2
Varying Grayscale Contrast
A.3
Fine and Contacting Particles
A.4
Intra-particle Porosity
Cropped
B
Pixel-wise Semantic Segmentation
2084 × 2084 pixel
500 µm
B.1
B.2
B.3
B.4
C
Particle Instance Segmentation
2084 × 2084 pixel
500 µm
C.1
C.2
C.3
C.4
